# Comparative Characterization of Gluten and Hydrolyzed Wheat Proteins

**DOI:** 10.3390/biom10091227

**Published:** 2020-08-24

**Authors:** Angelika Miriam Gabler, Katharina Anne Scherf

**Affiliations:** 1 Leibniz-Institute for Food Systems Biology at the Technical University of Munich, 85354 Freising, Germany; a.gabler.leibniz-lsb@tum.de; 2 Department of Bioactive and Functional Food Chemistry, Institute of Applied Biosciences, Karlsruhe Institute of Technology (KIT), 76131 Karlsruhe, Germany

**Keywords:** celiac disease, gel electrophoresis, gliadin, gluten, high-performance liquid chromatography (HPLC), hydrolyzed wheat proteins, wheat allergy

## Abstract

Hydrolyzed wheat proteins (HWPs) are widely used as functional ingredients in foods and cosmetics, because of their emulsifying and foaming properties. However, in individuals suffering from celiac disease or wheat allergy, HWPs may have a modified immunoreactivity compared to native gluten due to changes in molecular structures. Although a variety of HWPs are commercially available, there are no in-depth comparative studies that characterize the relative molecular mass (M_r_) distribution, solubility, and hydrophilicity/hydrophobicity of HWPs compared to native gluten. Therefore, we aimed to fill this gap by studying the above characteristics of different commercial HWP and gluten samples. Up to 100% of the peptides/proteins in the HWP were soluble in aqueous solution, compared to about 3% in native gluten. Analysis of the M_r_ distribution indicated that HWPs contained high percentages of low-molecular-weight peptides/proteins and also deamidated glutamine residues. We also found considerable differences between the seven HWPs studied, so that each HWP needs to be studied in detail to help explain its potential immunoreactivity.

## 1. Introduction

Wheat gluten is the viscoelastic mass that remains when starch and other water-soluble components are washed out of wheat dough [1]. According to Codex Standard 163–1987, wheat gluten consists of >80% protein, 5–10% lipids, and residues of starch and non-starch polysaccharides [2]. The term “gluten” describes a mixture of over 100 different proteins with a mono-, oligo-, or polymeric structure. Oligomeric and polymeric proteins consist of monomers linked by interchain disulfide bonds. Gluten proteins contain high amounts of glutamine (Gln/Glu: 37.1 mol-% in gliadins, 30.1 mol-% in glutenins) and proline (16.6 mol-% in gliadins, 11.9 mol-% in glutenins) as well as low amounts of amino acids with charged side chains, such as lysine (0.8 mol-% in gliadins, 2.1 mol-% in glutenins) [1,3]. According to the so-called Osborne fractionation, gluten proteins can be divided into two protein fractions depending on their solubility, the gliadins and the glutenins. Gliadins are soluble in 60% ethanol, whereas glutenins remain insoluble [4,5]. Furthermore, gluten proteins can be subdivided by sodium dodecyl sulfate-polyacrylamide gel electrophoresis (SDS-PAGE) or reversed-phase high-performance liquid chromatography (RP-HPLC) into gluten protein types: α-, γ-, ω1,2-, and ω5-gliadins and high- and low-molecular-weight glutenin subunits (HMW-GS and LMW-GS) [5,6,7].

Gliadins consist of mostly monomeric and glutenins of oligomeric and polymeric proteins. Gluten is responsible for the unique baking properties of wheat flour, by building a viscoelastic gluten network [1,7]. Wheat gluten is often used as an ingredient to improve and standardize the baking properties of wheat flours [1,8].

Furthermore, gluten is also used in various non-cereal products, in which the consumer generally does not expect gluten to be present. For example, wheat gluten is used as binding or protein-enriching agent in meat products or even cosmetics [9,10]. To improve wheat gluten solubility, it is treated in different chemical and biochemical ways, leading to modifications and/or partial hydrolysis. Deamidation of proteins takes place during hydrolysis with acid or alkali. In the case of enzymatic hydrolysis, the deamidation depends on the enzyme used [8,9,10]. Hydrolysis leads to the formation of proteins or peptides with lower relative molecular masses (M_r_) and deamidation to changes of the net charge. Both processes increase the solubility of gluten. Treatment with alkali removes intermolecular disulfide bonds, but usually, it does not lead to a cleavage of peptide bonds [8,11]. Sodium hydroxide is typically used for alkaline hydrolysis; hydrochloric acid or sulfuric acid for acidic hydrolysis; and enzymes like papain, trypsin, and pronase for enzymatic hydrolysis [8,12,13]. Furthermore, gluten can be modified by physical means, like high-pressure processing, heat treatment, extrusion, and UV irradiation [8,14,15].

Wheat is one of the most common foods that may cause adverse reactions, such as celiac disease, non-celiac gluten sensitivity, and wheat allergy. Celiac disease is a chronic small intestinal immune-mediated enteropathy in genetically predisposed individuals, caused by the ingestion of gluten. Its estimated prevalence is about 1% of the population worldwide, with 0.7% reported based on biopsy-confirmed cases and 1.4% reported based on seroprevalence [16]. Wheat allergy has a prevalence of 0.2–1% and is defined as an adverse immune response, with intestinal and extraintestinal symptoms occurring within minutes or hours after exposition [17]. Thereby, the exposition triggering the immunoglobulin E (IgE)-mediated allergy can be through inhalation, skin contact, or oral ingestion. Wheat allergy can be classified into immediate food allergy, wheat-dependent exercise-induced anaphylaxis (WDEIA), respiratory allergy, and skin allergy. Gluten proteins and inhibitors in wheat have been identified to play a key role in wheat allergy [17,18]. WDEIA is a cofactor-triggered wheat allergy and is subdivided into conventional-WDEIA (CO-WDEIA) and hydrolyzed wheat protein-WDEIA (HWP-WDEIA). In CO-WDEIA, patients react to native gluten proteins, whereas in HWP-WDEIA, patients are sensitized to HWP but tolerate native gluten. This underlines that hydrolysis affects the immunoreactivity of wheat proteins [19]. Allergic reactions to soap and cosmetics containing HWPs were reported [20,21]. Parts of the relevant immunoreactive epitopes are already pre-existent in native wheat protein aggregations and are laid bare through hydrolysis but not destroyed. Besides, new epitopes may be created through hydrolysis and simultaneous deamidation [20,21]. Additionally, the increased solubility of the HWP and the route of exposure also affects the immunoreactivity of HWP compared to the native form. Consequently, the degree and type of hydrolysis play an important role, because this could affect the transport through natural barriers, such as the small intestinal epithelium or the skin [19,20,21,22].

Gluten is used as the starting material for the production of HWP, which may also be called partially hydrolyzed gluten to better reflect this. There are various methods of how gluten can be hydrolyzed, including chemical, biochemical, and physical approaches, leading to highly variable preparations of HWP. In most cases, the functional properties of the HWP were described as emulsifying or foaming [8,23]. Additionally, the sensory properties were analyzed as well as the use of HWP as a nutritional additive. When characterizing the proteins and peptides in HWP, biochemical analyses, such as enzyme-linked immunosorbent assays (ELISAs) or Western blots, are widely used [23,24,25,26,27,28,29,30]. However, there are only few studies on commercially available HWP compared to native gluten. Gessendorfer et al. (2009) prepared three enzymatically hydrolyzed prolamins from wheat, rye, and barley and characterized the resulting HWP using ELISA, SDS-PAGE, and RP-HPLC [31]. Wieser and Scherf developed a well-characterized HWP for diagnosis and clinical investigations of wheat-related disorders [32], but comprehensive comparative investigations on commercially available HWP are missing. Additionally, the detection of modified gluten proteins can be done with ELISA or polymerase chain reaction (PCR) [9,33,34,35].

Considering the differences between HWP and native gluten in terms of immunoreactivity, it is reasonable to hypothesize that various treatments cause changes in the molecular structures of gluten. Consequently, there is a need to characterize commercial HWP in comparison to native gluten. In the present study, gluten and commercially available HWP products were analyzed for their crude protein contents, solubility, and M_r_ distribution of the proteins and peptides as well as the contents of free ammonium as an indicator for deamidation.

## 2. Materials and Methods

### 2.1. Reagents and Materials

All reagents and chemicals were from Merck (Darmstadt, Germany), Sigma-Aldrich (Darmstadt, Germany), Serva (Heidelberg, Germany), Applichem (Darmstadt, Germany), Carl Roth (Karlsruhe, Germany), Honeywell (Offenbach, Germany), and J.T. Baker (Arnhem, Netherlands) in analytical grade or higher. Ultra-pure water for HPLC was purified with an Arium 611VF water purification system (Sartorius, Goettingen, Germany). Prolamin Working Group (PWG)-gliadin was used for calibration, which is well suited to quantitate all types of different wheat proteins [5,7,36]. Wheat gluten and HWP were obtained from Hermann Kröner GmbH (Ibbenbueren, Germany), Sigma-Aldrich (Darmstadt, Germany), Mühle Schlingemann (Waltrop, Germany), Tereos (Lille, France), Solabia Group (Pantin, France) Manildra Group (Gladesville, Australia), Golden Peanut GmbH (Garstedt, Germany), Reform- und Muehlenbaeckerei Boesen GmbH (Langenfeld, Germany), and Tate & Lyle (Aalst, Belgium). Seven HWP (HWP 1–7), six gluten samples (G1–6), and two treated gluten samples (G7, G8) were purchased. G7 is described as denatured wheat protein with a high protein content and G8 as a slightly textured product from wheat protein. Protein/peptide markers used for the gel-permeation HPLC were purchased from Sigma-Aldrich (Darmstadt, Germany): β-amylase from sweet potato (200 kDa), albumin from bovine serum (66 kDa), carbonic anhydrase from bovine erythrocytes (29 kDa), cytochrome c from horse heart (12.4 kDa), α-lactalbumin (14 kDa), and glutathione (0.3 kDa). Furthermore, a gluten peptide (1.9 kDa) was received from Genescript Biotech (Piscataway Township, NJ, USA).

### 2.2. Determination of Crude Protein Contents

The crude protein content of all samples was determined by the combustion method according to Dumas (ICC Standard Method No.167). The samples were analyzed using a Leco TruSpec Nitrogen Analyzer (LECO, Mönchengladbach, Germany) to determine the nitrogen content after combustion at 950 °C. Ethylenediaminetetraacetic acid (EDTA) was used for calibration. The nitrogen content was multiplied by the factor 5.7 to calculate the crude protein content (wheat protein, ICC No. 105/2) [7].

### 2.3. Stepwise Fractionation According to Solubility

The stepwise fractionation according to solubility was performed according to the modified Osborne method by Wieser et al. (1998) [5]. Three fractions A, B, and C were received. Fraction A is soluble in aqueous salt solution (0.4 mol/L NaCl, 0.067 mol/L Na_2_HPO_4_/KH_2_PO_4_, pH 7.6), fraction B is soluble in 60% ethanol (*v*/*v*), and fraction C is soluble in 1-propanol/0.05 mol/L Tris/HCl, pH 7.5 (50% (*v*/*v*), with 2 mol/L (*w*/*v*) urea and 0.06 mol/L dithiothreitol, DTT) (glutenin extraction solution).

The gluten samples G1–G8 (20 mg) were first extracted with 3 × 0.5 mL aqueous salt solution to obtain fraction A by vortex mixing for 2 min, followed by stirring at room temperature for 30 min and centrifugation (25 min, 3750× *g*, 20 °C). The corresponding three supernatants each were united and filled up with the extraction solution to a volume of 2 mL. The residue was discarded. 

In a separate experiment, the gluten samples G1–G8 (20 mg) were extracted with 3 × 1.5 mL 60% ethanol (*v*/*v*) by 2 min vortex mixing, 30 min stirring at room temperature, and centrifugation (25 min, 3750× *g*, 20 °C). The three supernatants were combined, and the volume was adjusted to 5 mL with 60% ethanol. To calculate the content of fraction B, the content of fraction A was deducted from the 60% ethanol extract after the RP-HPLC measurement. To extract fraction C, the residue was extracted three times with 1.5 mL of glutenin extraction solution by vortex mixing for 2 min followed by stirring at 60 °C under nitrogen atmosphere for 30 min and centrifugation (25 min, 3750× *g*, 20 °C). The supernatants were united and filled up with the extraction solvent to a volume of 5 mL. All extracts were filtered (0.45 µm) and each sample was analyzed in triplicate (*n* = 3) [5,7].

HWP1–HWP7 were extracted three times with 0.5 mL aqueous salt solution to obtain fraction A. The residue was subsequently extracted three times with 0.5 mL 60% ethanol (*v*/*v*) to obtain fraction B. Then, the residue was extracted three times with 0.5 mL of solution C. All steps were carried out exactly as described above, except that the final volumes were 2 mL for fractions A, B, and C, respectively. All extracts were filtered (0.45 µm) and each sample was analyzed in triplicate (*n* = 3) [5,7].

### 2.4. Reversed-Phase High-Performance Liquid Chromatography (RP-HPLC)

Protein composition and contents in fractions A, B, and C were analyzed by RP-HPLC on a Jasco XLC instrument (Jasco, Gross-Umstadt, Germany) using a C_18_ column at 60 °C (Acclaim^TM^ 300, C_18_, 2.1 × 150 mm, 300 nm, 3 µm, Thermo Fisher Scientific, Braunschweig, Germany). The elution solvents were 0.1% trifluorocacetic acid (TFA) in ultra-pure water (A) and 0.1% TFA in acetonitrile (B). The flow rate was 0.2 mL/min with the following gradient: 0 min 0% B, 0.5 min 24% B, 20 min 56% B, 20.1–24.1 90% B, 24.2–30.0 min 0% B. 2) [5,7]. For G 1–8, the injection volume was 20 µL for fraction A and C and 10 µL for fraction B. For HWP 1–8, it was necessary to vary the injection volumes, because of the different protein contents in the fractions (1–20 µL). The absorbance at 210 nm was detected. PWG-gliadin was dissolved in 60% ethanol (*v*/*v*) to a concentration of 2.5 mg/mL and used for external calibration (50, 37.5, 25, 12.5, and 5 µg absolute) [36]. The limit of quantitation was estimated by injecting different amounts of PWG-gliadin (12.5, 5.00, 3.75, 2.50, 1.25, 0.50, and 0.25 µg absolute). The protein content of PWG-gliadin was 93.1 g/100 g. The software Jasco Chrompass was used for data analysis (version 1.2).

### 2.5. Gel-Permeation High-Performance Liquid Chromatography (GP-HPLC)

To analyze the M_r_ of proteins and peptides in HWP and treated gluten samples, HWP1–HWP7 and G7,8 were extracted by stepwise fractionation according to solubility. Three fractions I, II, and III were received. Fraction I, soluble in aqueous salt solution (0.4 mol/L NaCl, 0.067 mol/L Na_2_HPO_4_/KH_2_PO_4_, pH 7.6), corresponds to fraction A in Section 2.3. Fraction II, soluble in 60% ethanol, corresponds to fraction B in Section 2.3. Fraction III is a mixture of ultra-pure water and acetonitrile (50/50, *v*/*v*) and corresponds to fraction C in Section 2.3. Each fraction was extracted three times. Fraction I and II were extracted from HWP (20.0 mg or 100 mg) as described for fractions A and B in Section 2.3 for HWP. Fraction III was extracted from the residue three times with 0.5 mL of ultra-pure water and acetonitrile (50/50, *v*/*v*) by vortex mixing for 2 min followed by stirring at room temperature for 30 min and centrifugation (25 min, 3750× *g*, 20 °C). The corresponding three supernatants were united and filled up with the respective extraction solvent to a volume of 2 mL. All extracts were filtered (0.45 µm) and each sample was analyzed in triplicate (*n* = 3) [5].

For chromatographic analysis, two GP-HPLC systems were used. For larger molecules, a GP-HPLC system according to Scherf et al. (2016) was used (system 1) [36]. A BioSep-SEC-s3000 (300 × 4.6 mm, 29 nm, 5 μm, Phenomenex) was used on a Jasco HPLC Extrema (Jasco, Gross-Umstadt, Germany) at 20 °C. Elution solvents A and B were used with a flow rate of 0.3 mL/min and an isocratic gradient composition of 50% A and 50% B. Because of different protein/peptide contents in the different fractions, the injection volumes were between 1 and 20 µL. The absorbance at 210 nm was detected. To separate smaller molecules, a second GP-HPLC system was used with a BioBasic SEC-60 column (150 × 7.8 mm, 6 nm, 5 μm, Thermo Scientific) on the same Jasco HPLC Extrema at 20 °C (system 2). The elution solvents were 0.1% TFA in ultra-pure water (A) and 0.1% TFA in acetonitrile (B). The flow rate was 1 mL/min with an isocratic gradient composition of 70% A and 30% B for a duration of 12 min. Because of the different protein/peptide contents in the different fractions, the injection volumes were between 5 and 20 µL. 

The absorbance at 210 nm was detected [37]. The software ChromNAV was used for data analysis (Jasco Deutschland GmbH, Pfungstadt, Germany). Proteins and peptides with known M_r_ were measured to mark integration areas for a certain M_r_ range. β-Amylase from sweet potato (200 kDa), albumin from bovine serum (66 kDa), carbonic anhydrase from bovine erythrocytes (29 kDa), and cytochrome c from horse heart (12.4 kDa) were used for system 1. α-Lactalbumin (14.2 kDa), a gluten peptide (1.9 kDa), and glutathione (0.3 kDa) were used for system 2. The area under the curve (AUC) was integrated in each section and calculated as the percentage of the total area.

### 2.6. Sodium Dodecyl Sulfate-Polyacrylamide Gel Electrophoresis (SDS-PAGE)

SDS-PAGE was performed according to Lagrain et al. (2012) [6]. G1–G8 and HWP1–HWP7 were characterized by SDS-PAGE on a homogenous NuPAGE 10% polyacrylamide Bis-Tris gel (10 × 1 mm wells) (Invitrogen, Carlsbad, CA, USA). A mixture of proteins was used as a size standard (PageRuler^TM^ Unstained Protein Ladder, Thermo Fisher Scientific). All samples (1.5 mg each) were mixed with 1 mL of extraction buffer (200 g/L sucrose, 59.7 g/L Tris-HCl, 40 g/L SDS, 0.3 g/L EDTA, 0.4 g/L Coomassie blue, 0.1 g/L phenol red, 0.11 mmol/L HCl) containing 7.72 g/L DTT. After incubation for 12 h, the samples were heated to 60 °C for 10 min and centrifuged (5 min, 5000× *g*, 20 °C). Two different running buffers were used. The MOPS running buffer consisted of 20.9 g/L 3-(N-morpholino) propane sulfonic acid (MOPS), 12.1 g/L Tris-HCl, 2 g/L SDS, 0.6 g/L EDTA, and 0.77 g/L DTT as the reducing agent. The MES running buffer consisted of 20.9 g/L 2-(N-morpholino) ethane sulfonic acid (MES), 12.1 g/L Tris-HCl, 2 g/L SDS, 0.6 g/L EDTA, and 0.77 g/L DTT, as the reducing agent. The running time was 30 min at 115 mA and 200 V. The protein bands were fixed with 12% (*w*/*w*) trichloroacetic acid for 30 min, stained with Coomassie blue for 30 min, and destained first with methanol/glacial acetic acid/water (50/10/40, *v*/*v*/*v*), and then with methanol/glacial acetic acid/water (10/10/80, *v*/*v*/*v*). The gels were scanned using the Gel Doc^TM^ EZ Imager (Bio-Rad Laboratories, Munich, Germany) and the Image Lab software (Bio-Rad Laboratories, Munich, Germany). The images were converted to grayscale [6,7].

### 2.7. Contents of Free Ammonium

For the determination of free ammonium in G1–G8 and HWP1–HWP7, 50 mg of sample (*n* = 3) were weighed into a 50-mL two-neck round-bottom flask with a 25-mL dropping funnel with a vacuum equalizer and a vacuum receiver. Glass wool was inserted into the vacuum receiver and wetted with 200 µL sulphuric acid (0.5 mol/L). Vacuum was created using a vacuum pump and 5 mL of boric acid/sodium hydroxide buffer (pH 10) were added via the dropping funnel. Ammonia was expelled for two hours while stirring at room temperature. The glass wool was then transferred into a 50-mL volumetric flask and the vacuum receiver was washed with water. The measurement was performed by photometry [38]. For this purpose, the samples were mixed with 4 mL each of the following solutions: (a) 130 g/L sodium salicylate, 130 g/L trisodium citrate dihydrate, and 970 g/L disodium-tacanonitrosyl-(III)-ferrate-dihydrate dissolved in water; (b) 0.032 g/L sodium hydroxide and 0.002 g/L sodium dichloro-isocyanurate dissolved in water. The flask was then filled up with water to a volume of 50 mL and left to stand for 1 h at room temperature. The measurement was carried out according to DIN ISO 11732 with a UV-2401 spectrophotometer PC (Shimadzu, Neufahrn, Germany) at 655 nm. An external calibration line was prepared using ammonium sulfate [38].

### 2.8. Statistical Analysis

Statistical analysis was carried out with the use of Origin 19 (OriginLab Cooperation, Northampton, MA, USA) and SigmaPlot 14 (Systat Software GmbH, Erkrath, Germany). 

## 3. Results

### 3.1. Determination of Crude Protein Contents

The untreated (G1–G6) and the treated gluten samples (G7–G8) showed comparable crude protein contents with 703.2–855.1 and 731.6–765.0 mg/kg, respectively (Table 1). The HWP had a wider range and HWP5 (650 mg/g) had the lowest and HWP7 (898 mg/g) the highest crude protein content. Still, the mean values of gluten samples and HWP were quite similar (G1–G8: 773 ± 45.3 mg/g; HWP1–HWP7: 756 ± 67.9 mg/g; total: 765 ± 57.6 mg/g). HWP5 and G4 were significantly different from each other.

### 3.2. Stepwise Fractionation According to Solubility and RP-HPLC

**Figure 1:**
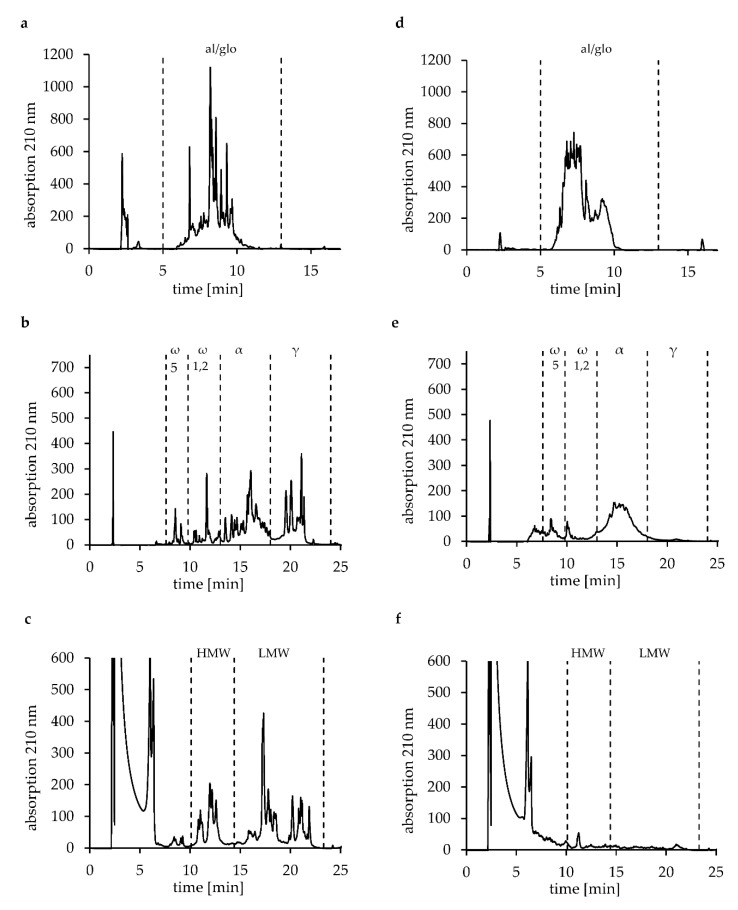
Reversed-phase high-performance liquid-chromatography: Chromatograms of the three fractions, soluble in aqueous salt solution (fraction A), soluble in 60% ethanol (fraction B), and soluble in glutenin extraction solution (fraction C) of gluten G1 (**a**–**c**) and hydrolyzed wheat protein HWP2 (**d**–**f**). Albumins and globulins (fraction A: **a**,**d**), gliadins subdivided into ω5-, ω1,2-, α-, and γ-gliadins (fraction B: **b**,**e**), and glutenins subdivided into high-molecular-weight- (HMW-) and low-molecular-weight- (LMW-) glutenin subunits (fraction C: **c**,**f**).

The contents of fractions A, B, and C in gluten samples and HWP were determined according to the modified Osborne fractionation, combining extraction and RP-HPLC analysis [4,7]. The RP-HPLC chromatograms of the three fractions from gluten (G1) and HWP2 are shown as examples in Figure 1 and those of all other samples are available as Supplementary Material Figures S1–S13. Fraction B consists of gliadins, whereas fraction C contains glutenins. The contents of fraction B were between 321.3 and 505.6 mg/g and fraction C between 182.4 and 331.3 mg/g. The contents of fraction B in G3, G7, and G8 were significantly different to each other and to other gluten samples as well as HWP (Table 1). Residues of albumins and globulins, present in fraction A, were determined (11.0–21.6 mg/g). They are mostly washed out during the gluten extraction process of wheat gluten but not completely. The content of fraction A was similar for gluten samples in most cases. The contents of the three fractions were similar for G8 (A: 10.9 mg/g, B: 183.7 mg/g, C: 336.9 mg/g) compared to the untreated gluten samples (G1–G6). G7 (A: 14.8 mg/g, B: 321.3 mg/g, C: 331.3 mg/g) had a similar content of fraction C but significantly different contents of fraction A and B in relation to G1–G6 and G8 and all HWP (except fraction A of G2).

The contents of fraction A from all HWP (45.8–569.4 mg/g) were significantly higher compared to gluten G1–G8. This results from the modification process where gluten proteins are degraded to smaller proteins, which are consequently more soluble in aqueous solutions. Furthermore, the contents of fraction A from HWP1–HWP7 were significantly different to each other. For HWP, the contents were between 25.9 and 423.1 mg/g for fraction B and between 15.4 and 162.2 mg/g for fraction C. For HWP1 and HWP3, the contents of fraction B were lower than 2.3 mg/g. This value was estimated by injecting decreasing amounts of PWG-gliadin and checking the linearity of the calibration curve. For HWP1, HWP2, and HWP3, the contents of fraction C were lower than 4.7 mg/g. HWP2 and HWP5 had a significantly lower content of fraction B than the other HWP and G1–G8. 

### 3.3. Gel-Permeation High-Performance Liquid Chromatography (GP-HPLC)

**Figure 2:**
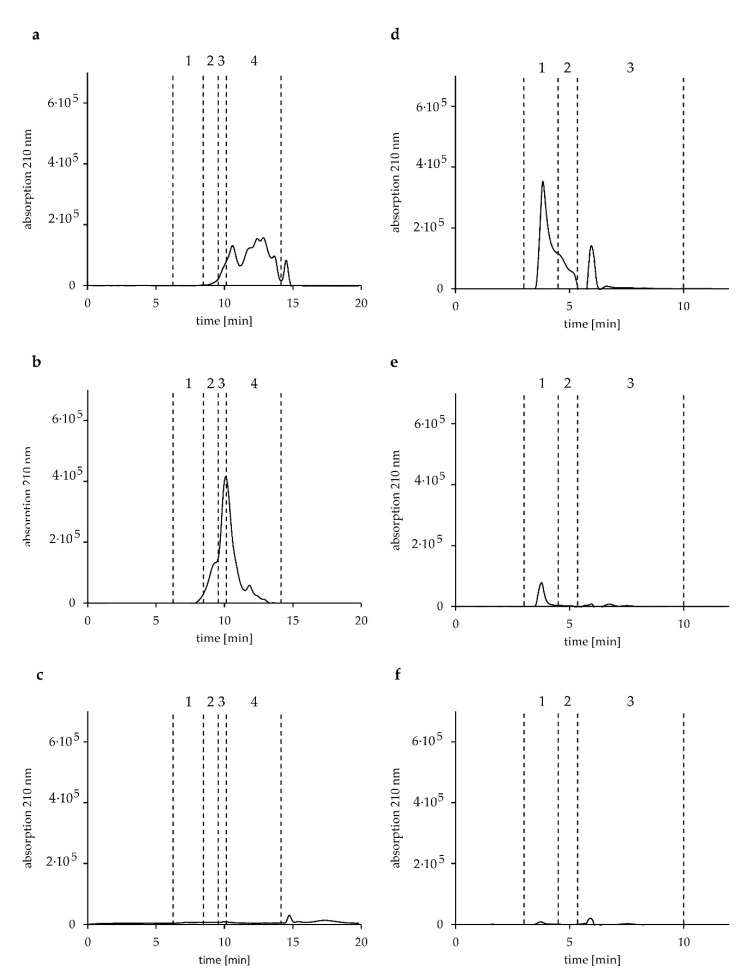
Gel-permeation high-performance liquid-chromatography: Chromatograms of the three fractions, soluble in aqueous salt solution (fraction I: **a**,**d**), soluble in 60% ethanol (fraction II: **b**,**e**), and soluble in acetonitrile/water (50/50, *v*/*v*) (fraction III: **c**,**f**) of HWP2 in two different systems. System I (**a**–**c**) is subdivided into the following ranges of relative molecular masses: M_r_ 200–66 kDa (1), M_r_ 66–29 kDa (2), 29–14 kDa (3), < 14 kDa (4). System II (**d**–**f**) is subdivided into the following ranges of relative molecular masses: M_r_ ≥ 14 kDa (1), M_r_ 14–2 kDa (2), M_r_ < 2 kDa (3).

To have a closer look at the different HWP and modified gluten samples, fractions I, II, and III were analyzed by GP-HPLC to determine the M_r_ distribution of the proteins and peptides using two chromatographic systems (I and II). After the extraction of fractions I and II according to the modified Osborne fractionation, fraction III was extracted with a water-acetonitrile mixture (50/50, *v*/*v*). It was impossible to use the glutenin extraction solution, as commonly used in the modified Osborne fractionation combined with RP-HPLC, because it generated a broad interfering signal in the GP-HPLC chromatogram at a retention time of 6.9 min in system I. The chromatograms of the three fractions analyzed by the two GP-HPLC systems are shown in Figure 2 for HWP2 and the quantitative results are presented in Table 2 and Table 3. The GP-HPLC chromatograms of all other HWP samples and of G7 and G8 are available as Supplementary Material Figures S14–S29. 

HWP1 and HWP3 both contained only proteins/peptides with M_r_ below 14 kDa in fraction I. No peaks were detectable anymore in fractions B and C of the modified Osborne fractionation (Table 1) and consequently, fractions II and III in the GP-HPLC system I also showed no AUC. This clearly shows that HWP1 and HWP3 were extensively hydrolyzed. The M_r_ distribution of HWP2 showed 92.0% of proteins/peptides with an M_r_ below 14 kDa, 6.5% with an M_r_ of 14–29 kDa, and 1.5% with an M_r_ of 66–29 kDa in fraction I. In fraction II, the distribution of M_r_ changed, but 50.4% of the proteins/peptides were still below 14 kDa. No signals were detected in fraction III of HWP2, as expected from fraction C. All other HWP4–HWP7 as well as G7 and G8 displayed signals above the estimated threshold in all three fractions (Table 2). HWP5 and HWP6 showed quite similar M_r_ distributions in fractions I, II, and III (Table 2). HWP4 and HWP7 contained no proteins/peptides with M_r_ 200–66 kDa in fraction I. Furthermore, they had low percentages for M_r_ 29–66 kDa (2.3–4.0%) and M_r_ 14–29 kDa (5.0–8.8%) but high percentages for M_r_ < 14 kDa (87.2–92.7%) in fraction I. Both fractions II and III of HWP7 had similar percentages in M_r_ as HWP6. Fraction II of HWP4 showed a different M_r_ distribution compared to the other samples, whereas fraction III was comparable to HWP6 and HWP7. The treated gluten samples G7 and G8 were comparable in the percentages of M_r_ in all fractions.

The GP-HPLC system II was established to have a closer look at proteins/peptides with low M_r_ (Table 3). As expected from the Osborne fractionation (Table 1), HWP1 and HWP3 only showed baseline values in fractions II and III. The M_r_ distribution in fraction I of HWP1 and HWP3 was significantly different to all other samples and to each other. HWP1 had significantly higher percentages of proteins/petides with M_r_ ≥ 14 kDa but lower percentages of proteins/petides with M_r_ < 2 kDa compared to HWP3. In fraction I, the M_r_ of HWP2 was mostly ≥ 14 kDa (66.3%), whereas the percentage of peptides with an M_r_ < 2 kDa was similar to that of HWP5. HWP4 and HWP5 as well as HWP6 and HWP7 showed comparable M_r_ distributions in fraction I, respectively. Considering fractions II and III, HWP1 and HWP3 only had baseline values, indicating that the samples were completely soluble in aqueous salt solution. HWP2 and HWP5 showed no peaks in fraction III, but fraction II contained 100% of proteins/peptides with M_r_ ≥ 14 kDa. 

When comparing the results from RP-HPLC and GP-HPLC analyses, we found 15.4 mg/g of fraction C in HWP5 after extraction/RP-HPLC analysis (Table 1) but no signal above the baseline after extraction/GP-HPLC analysis. This discrepancy can be explained with the change of extraction solvents for both analyses. Apparently, the glutenin extraction solution with DTT used in combination with RP-HPLC (fraction C) is more efficient at extracting the proteins/peptides from the sample than the water-acetonitrile mixture used for GP-HPLC (corresponding fraction III). The difference was small and only apparent for HWP5, because it had such low contents of fraction C/III. HWP4, HWP6, and HWP7 had 100% of proteins/peptides with M_r_ ≥ 14 kDa in fractions II and III, respectively, as did G7 and G8. The M_r_ distribution of G7 and G8 was significantly different from HWP1–HWP7 in fraction I. G8 was the only sample with no AUC in the range of M_r_ 14–2 kDa. G7 and G8 were also significantly different to each other, which is according to expectations, because they were treated in different ways.

### 3.4. Sodium Dodecyl Sulfate-Polyacrylamide Gel Electrophoresis (SDS-PAGE)

**Figure 3:**
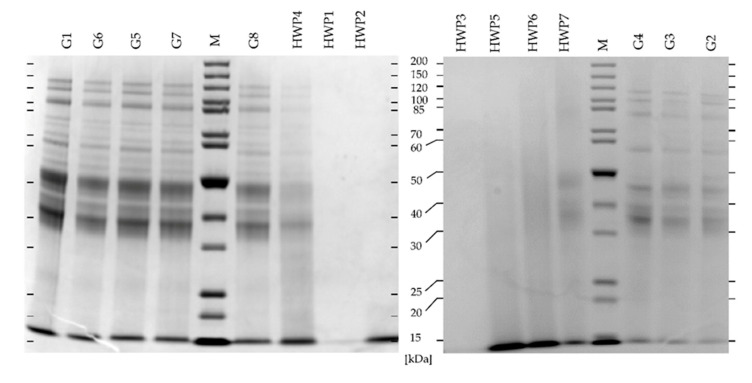
Sodium dodecyl sulfate-polyacrylamide gel electrophoresis of gluten samples (G1–G8) and hydrolyzed wheat proteins (HWP1–HWP7). Protein marker 3.5 µg, samples 15 µg.

Two different reducing buffer systems, using MES and MOPS, were tested for SDS-PAGE. Overall, the gels looked similar regarding band patterns, but the bands were sharper with MOPS compared to MES. Consequently, the SDS-PAGE gels with MOPS running buffer are discussed in the following (Figure 3). The protein marker contained proteins with 15 and 10 kDa, but these bands were not separated at the end of the gel, so that all bands in this range were designated as M_r_ ≤ 15 kDa. 

Gluten protein types have different M_r_, ranging from HMW-GS with 65–90 kDa, to LMW-GS with 30–50 kDa and to gliadins with 28–55 kDa. Among gliadins, the M_r_ ranges are 49–55 kDa for ω5-gliadins, 39–44 kDa for ω1,2-gliadins, and 28–39 kDa for α- and γ-gliadins. Veraverbeke et al. and Lagrain et al. reported that the M_r_ of HMW-GS is overestimated to 80–120 kDa in SDS-PAGE due to aggregation effects This is visible in all gluten samples (G1–G8), with three characteristic bands of HMW-GS in this range. In general, the native gluten samples (G1–G6) showed the typical protein bands of the different gluten protein types. A protein band with M_r_ ≤ 15 kDa was also present in each native gluten sample (G1–G6), which results from residues of albumins and globulins (see also Section 3.2, fraction A) [1,6,7,39].

The treated gluten samples G7 and G8 showed the same protein bands as the native gluten samples G1–G6, indicating that the treatment did not cause extensive changes in the protein composition. In contrast, the HWP were completely different. Depending on their degree of hydrolysis, they showed more or fewer protein bands. HWP4 and HWP7 showed protein bands in the range of 30–50 kDa and approximately 60 and 85 kDa, but they were much more blurred compared to G1–G8. HWP5 and HWP6 showed only blurred lanes with no discernible protein bands. HWP2, HWP5, and HWP6 showed one band at M_r_ ≤ 15 kDa, which was also visible in G1–G8, as well as HWP4 and HWP7. HWP1 and HWP3 showed no protein bands at all, indicating that they had been extensively hydrolyzed.

### 3.5. Contents of Free Ammonium

The content of free ammonium in a sample is an indicator for hydrolysis under drastic conditions, like highly concentrated mineral acids and high temperature, or the use of deamidating enzymes, such as transglutaminases [9]. The native gluten samples (G1–G6) had low contents of free ammonium (0.05–0.12 mg/g) (Table 1), as did the treated gluten samples (G7–G8: 0.10 mg/g). However, the values were higher (0.13–5.00 mg/g) for HWP. This was expected and depends on the production process. HWP1 and HWP3 had the highest contents of free ammonium (4.12 and 5.00 mg/g, respectively). G2 and HWP3 had significantly different contents of free ammonium.

As shown with RP-HPLC, GP-HPLC, and SDS-PAGE, HWP1 and HWP3 were both extensively hydrolyzed wheat protein samples. These two samples may have been processed via hydrolysis with mineral acid under heating, which is a very common procedure in the food industry. As shown above, HWP2 has also been extensively hydrolyzed. In contrast to HWP1 and HWP3, the ammonium content of HWP2 was low (0.59 mg/g) and this could be an indicator that HWP2 might have been hydrolyzed enzymatically, with a long duration [9].

## 4. Discussion

Gluten and HWP showed similarities in the crude protein contents (about 765 mg/g) and these contents were expected for gluten [1]. This shows that the treatment of the HWP did not change the crude protein content, except for HWP 5. The content of free ammonium was used as an indicator for deamidation. Consequently, higher values were expected for the HWP compared to gluten and the results supported the expectation, because HWP had average contents of 1.56 mg/g and gluten of 0.09 mg/g. No exact contents have been reported in the literature so far, but it is known that deamidation may take place during hydrolysis and is also carried out intentionally to achieve the desired functional properties, such as increased solubility [8,9].

The most remarkable differences between gluten and HWP were the contents of fraction A of the modified Osborne fractionation. HWP (on average: 341.6 mg/kg) showed significantly higher contents than gluten (on average: 16.0 mg/kg), according to expectations. Crude gluten is treated in chemical and biochemical ways to improve solubility. Consequently, a higher percentage of proteins or peptides is soluble in salt solution and does not require organic solvents or reducing agents anymore to become soluble, like intact gliadins and glutenins do. The extent of the increase in solubility and thus contents of fraction A depend on the type and degree of processing. Consequently, with increased contents in fraction A, HWP had decreased contents in fraction B and C. Among others, Kanerva et al. (2011) and Wu et al. (1976) described a noticeably increased solubility of HWP compared to gluten [9,28].

In agreement with the RP- and GP-HPLC results, differences were also visible between gluten and HWP using SDS-PAGE. While gluten showed typical protein bands, the HWP showed less or even no protein bands, because of protein degradation. Generally, the protein bands were weaker in HWP than in gluten, which was expected. Wieser et al. (2018) showed a change in protein bands in SDS-PAGE relative to the duration of hydrolysis [32]. However, the degree of hydrolysis and the presence of protein bands in SDS-PAGE is not only dependent on the duration but also on the type of hydrolysis. Chemical hydrolysis is usually harsher than enzymatic digestion [8,12,32,40].

Interestingly, in SDS-PAGE, a protein band was found at M_r_ ≤ 15 kDa in G1–G8 and in most HWP samples, except HWP1 and HWP3. In the GP-HPLC system I, this is also clearly visible in area 4 of fraction I (M_r_ < 14 kDa), which had the highest percentage in the fraction of these samples. Additionally, in the GP-HPLC system II, the highest percentage was present in area 1 of fraction I (M_r_ ≥14 kDa) for HWP2, 4–7 and G7. This indicates the presence of proteins with M_r_ around about 14 kDa or lower. This may result from, e.g., α-amylase/trypsin-inhibitors that have a M_r_ of about 12–16 kDa, but further analyses are required to unambiguously identify these proteins [41]. 

HWP1 and HWP3 did not show any protein bands in SDS-PAGE, which indicated the status of total hydrolysis. This is also visible in their GP-HPLC measurements, showing M_r_ lower than 14 kDa in both systems. Such an extensive hydrolysis can be carried out with the use of 0.5–1 mol/L hydrochloric acid and boiling [9,28].

Furthermore, the HWP were different from each other in many cases. The contents of fractions A and B were significantly different in every HWP. Additionally, differences in M_r_ using GP-HPLC were visible. It was predictable that HWP were different from each other, because many different approaches for gluten hydrolysis are in use like treatment with chemicals [13] and enzymes or high-pressure processing and UV irradiation [8].

The differences between native gluten and HWP are likely to result in difficulties regarding gluten analysis, important for people with wheat-related disorders, who need to avoid gluten. The identified differences between HWP support the allegation that their determination is challenging [9,33,35]. 

In general, it should be noted that the differences between gluten and HWP need to be considered when developing analytical methods, e.g., because sample preparation is affected due to different solubility. Another point is that reference materials used for calibration may need to be adapted or that different assay formats may be necessary, i.e., a competitive ELISA as opposed to a sandwich format [31,35]. Regarding diagnostic approaches, the immunoreactivity might differ greatly, as the differences found at the molecular level suggest. The same applies to different HWP, because their properties are highly variable. The in-depth characterization of the samples allowed us to select particularly interesting HWP samples showing a low or a high degree of hydrolysis for further work to characterize the sensitization profiles in wheat allergic patients. Testing the levels of gluten immunogenic peptides arising after the ingestion of gluten or HWP in patients’ urine or stool samples would be very interesting to assess potential differences in bioaccessibility, bioavailability, and uptake vs. excretion ratios [42].

How the identified molecular differences influence the mechanisms of celiac disease and wheat allergy is hard to say. On the one hand, hydrolysis uncovers immunoactive epitopes in the proteins and potentially generates new ones by deamidation. In addition, the increased solubility of HWP may have an influence on the bioavailability and digestibility in the body. On the other hand, hydrolysis can also destroy immunoactive epitopes, because of extensive protein degradation [19,20,21,22]. 

## 5. Conclusions

Commercially available HWP and gluten samples were characterized according to their crude protein content, solubility, and M_r_ of the proteins and peptides as well as the content of free ammonium as indicator for deamidation. Differences in the protein composition, solubility, and M_r_ distribution between HWP and native gluten were expected and found, especially for the solubility of HWP and gluten in aqueous salt solution. Additionally, all analyzed HWP were significantly different from each other. This shows that the molecular characteristics of HWP generally are highly variable and that these are likely to cause differences in the immunoreactivity of the products. These findings highlight that the exact characterization of HWP products is very important to establish relationships between protein structure and immunoreactivity for patients suffering from wheat-related disorders. It is necessary to pay attention to the molecular differences between gluten and HWP, especially for the development of analytical or diagnostic methods.

## Figures and Tables

**Table 1:** Contents of fractions A, B, and C and their sum (SUM), crude protein and free ammonium in the gluten samples (G1–G8) and hydrolyzed wheat proteins (HWP1–HWP7). Values are given as means (*n* = 3) and different capital letters indicate significant differences between the samples in each column (one-way ANOVA, Tukey’s post hoc test, *p* < 0.05).

Sample	Fraction A	Fraction B	Fraction C	SUM	Protein Content	Free NH_4_^+^
	[mg/g]	[mg/g]	[mg/g]	[mg/g]	[mg/g]	[mg/g]
HWP 1	400.7 ^G^	<2.3	<4.7	400.7	737.4 ^A,B^	4.12 ^A,B^
HWP 2	569.4 ^J^	25.9 ^A^	<4.7	595.3	752.6 ^A,B^	0.59 ^A,B^
HWP 3	493.6 ^I^	<2.3	<4.7	493.6	764.9 ^A,B^	5.00 ^B^
HWP 4	158.2 ^E^	237.2 ^E^	142.5 ^B^	537.9	737.3 ^A,B^	0.39 ^A,B^
HWP 5	443.4 ^H^	118.3 ^B^	15.4 ^A^	577.1	649.9 ^A^	0.13 ^A,B^
HWP 6	280.0 ^F^	150.2 ^C^	162.2 ^B,C^	592.4	750.2 ^A,B^	0.15 ^A,B^
HWP 7	45.8 ^D^	423.1 ^G^	146.5 ^B^	615.4	898.8 ^B^	0.56 ^A,B^
G 1	18.4 ^C^	410.9 ^G^	191.8 ^C,D^	621.1	766.2 ^A,B^	0.11 ^A,B^
G 2	15.1 ^B^	509.6 ^I^	226.6 ^D^	751.3	824.5 ^A,B^	0.05 ^A^
G 3	14.8 ^C^	460.6 ^H^	212.9 ^D^	688.3	778.1 ^A,B^	0.12 ^A,B^
G 4	11.0 ^A^	505.6 ^I^	212.9 ^D^	729.5	855.1 ^B^	0.05 ^A^
G 5	21.1 ^C^	394.2 ^G^	182.4 ^C^	597.7	757.3 ^A,B^	0.09 ^A,B^
G 6	21.6 ^C^	427.6 ^G^	208.9 ^D^	658.1	703.2 ^A,B^	0.11 ^A,B^
G 7	14.8 ^B^	321.3 ^F^	331.3 ^E^	667.4	765.0 ^A,B^	0.10 ^A,B^
G 8	10.9 ^A^	183.7 ^D^	336.9 ^E^	531.5	731.6 ^A,B^	0.10 ^A,B^
VC *	2.24	2.29	3.13	-	6.14	5.14

* Variation coefficient [%]: median of all relative standard deviations per analysis.

**Table 2:** Gel-permeation HPLC (system I): Analysis of fractions I, II, and III of treated gluten (G7 and G8) and hydrolyzed wheat proteins (HWP1–HWP7) according to their relative molecular mass distribution. Areas within each fraction were set by marker substances. 1: 200–66 kDa; 2: 66–29 kDa; 3: 29–14 kDa; 4: < 14 kDa. Values are given as means (*n* = 3) and different capital letters indicate significant differences between the samples in each column (one-way ANOVA, Tukey’s post hoc test, *p* < 0.05). “-” integration not possible, because the area under the curve was not different to baseline.

Sample	Fraction I				Fraction II				Fraction III			
	1	2	3	4	1	2	3	4	1	2	3	4
	[%]	[%]	[%]	[%]	[%]	[%]	[%]	[%]	[%]	[%]	[%]	[%]
HWP 1	-	-	-	100.0 ^B^	-	-	-	-	-	-	-	-
HWP 2	-	1.5 ^A^	6.5 ^D^	92.0 ^A,B^	-	17.0 ^B^	32.6 ^A,B,C^	50.4 ^C^	-	-	-	-
HWP 3	-	-	-	100.0 ^B^	-	-	-	-	-	-	-	-
HWP 4	-	2.3 ^A,B^	5.0 ^C^	92.7 ^A,B^	14.0 ^C^	16.1 ^A,B^	26.2 ^A,B,C^	43.7 ^B,C^	60.2 ^A,B^	14.3 ^A,B^	9.8 ^A,B^	15.7 ^A,B^
HWP 5	11.6 ^A^	10.6 ^B^	11.6 ^F^	66.2 ^A^	39.5 ^F^	14.2 ^A^	9.7 ^A^	36.6 ^A,B,C^	52.0 ^A,B^	14.3 ^A,B^	8.3 ^A,B^	25.4 ^A,B^
HWP 6	7.3 ^B^	9.7 ^A,B^	15.6 ^G^	67.4 ^A^	31.6 ^E^	15.2 ^A,B^	16.4 ^A,B^	36.8 ^A,B,C^	63.5 ^B^	13.4 ^A,B^	7.8 ^A^	15.3 ^A,B^
HWP 7	-	4.0 ^A,B^	8.8 ^E^	87.2 ^A,B^	31.0 ^D,E^	16.9 ^B^	24.6 ^A,B,C^	27.5 ^A^	61.3 ^A,B^	12.2 ^A^	11.7 ^A,B^	14.8 ^A^
G 7	-	-	3.5 ^B^	96.5 ^A,B^	11.3 ^B^	15.1 ^A,B^	39.5 ^B,C^	34.1 ^A,B^	30.7 ^A,B^	12.1 ^A^	37.6 ^A,B^	19.6 ^A,B^
G 8	-	-	2.0 ^A^	98.0 ^A,B^	5.8 ^A^	14.7 ^A,B^	42.9 ^C^	36.6 ^A,B,C^	11.7 ^A^	15.3 ^B^	45.0 ^B^	28.0 ^B^
CV * [%]	8.8	4.9	4.9	0.7	2.8	1.3	3.6	2.1	3.3	2.3	6.5	5.5

* Variation coefficient: median of all relative standard deviations per analysis.

**Table 3:** Gel-permeation HPLC (system II): Analysis of fractions I, II, and III of treated gluten (G7 and G8) and hydrolyzed wheat proteins (HWP1-HWP7) according to their relative molecular mass distribution. Areas within each fraction were set by marker substances. 1: ≥ 14 kDa; 2: 14–2 kDa; 3: < 2 kDa. Values are given as means (*n* = 3) and different capital letters indicate significant differences between the samples in each column (one-way ANOVA, Tukey’s post hoc test, *p* < 0.05). “-” integration not possible, because the area under the curve was not different to baseline.

Sample	Fraction I			Fraction II			Fraction III		
	1	2	3	1	2	3	1	2	3
	[%]	[%]	[%]	[%]	[%]	[%]	[%]	[%]	[%]
HWP 1	16.8 ^B^	33.9 ^G^	49.3 ^F^	-	-	-	-	-	-
HWP 2	66.3 ^E^	18.8 ^F^	14.9 ^B^	100.0	-	-	-	-	-
HWP 3	3.9 ^A^	39.1 ^H^	57.0 ^G^	-	-	-	-	-	-
HWP 4	75.3 ^I^	13.7 ^E^	11.0 ^A^	100.0	-	-	100.0	-	-
HWP 5	73.7 ^G,H,I^	11.5 ^D^	14.8 ^B^	100.0	-	-	-	-	-
HWP 6	74.2 ^H,I^	4.5 ^B,C^	21.3 ^C^	100.0	-	-	100.0	-	-
HWP 7	70.5 ^F^	5.6 ^C^	23.9 ^D^	100.0	-	-	100.0	-	-
G 7	53.3 ^D^	2.5 ^A^	44.2 ^E^	100.0	-	-	100.0	-	-
G 8	31.6 ^C^	-	68.4 ^H^	100.0	-	-	100.0	-	-
CV * [%]	2.0	2.9	2.6	0.0	-	-	0.0	-	-

* Variation coefficient: median of all relative standard deviations per analysis.

## Supplementary Materials

The following are available online at https://www.mdpi.com/2218-273X/10/9/1227/s1, Figures S1–S13: Reversed-phase HPLC analysis of G2 to G8 as well as HWP1 and HWP3 to HWP7, Figure S14–S21: Gel-permeation HPLC analysis of HWP1, HWP3 to HWP7 and G7 and G8, Figure S22–S29: Gel-permeation HPLC analysis of HWP1, HWP3 to HWP7 and G7 and G8.
