# Supplementary material for: Comparative Characterization of Gluten and Hydrolyzed Wheat Proteins
Source: Biomolecules. 2020 Aug 24;10(9):1227. doi: 10.3390/biom10091227 (PMC7564556; doi:10.3390/biom10091227)
Supplement: Supplementary file 1 [file biomolecules-10-01227-s001.pdf]

Gabler AM, Scherf KA, Comparative characterization of gluten and hydrolyzed wheat proteins

Supplementary Figures

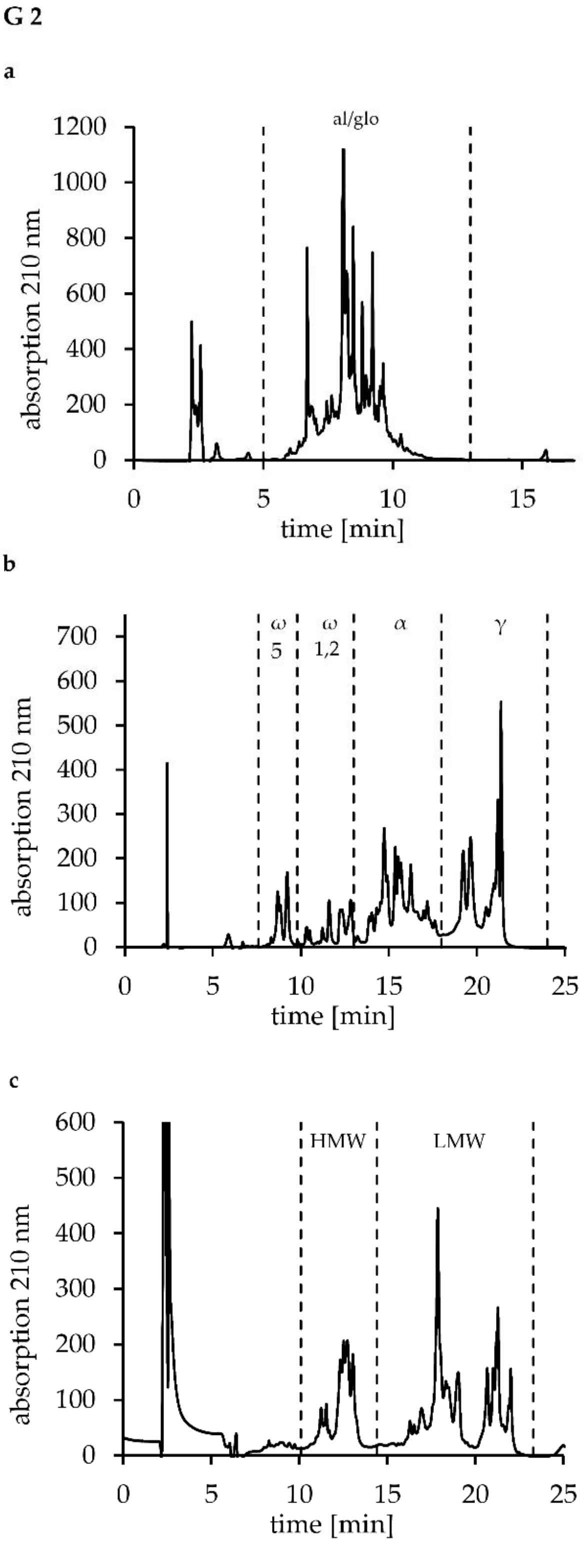

Supplementary Figure S1. Reversed-phase HPLC analysis of G2. Fraction A, albumins and globulins, soluble in aqueous salt solution (**a**), fraction B, gliadins soluble in 60% aqueous ethanol and subdivided into ω5-, ω1,2-, α- and γ-gliadins (**b**) and fraction C, glutenins, soluble in glutenin extraction solution subdivided into high-molecular-weight- (HMW-) and low-molecular-weight- (LMW-) glutenin subunits (**c**).

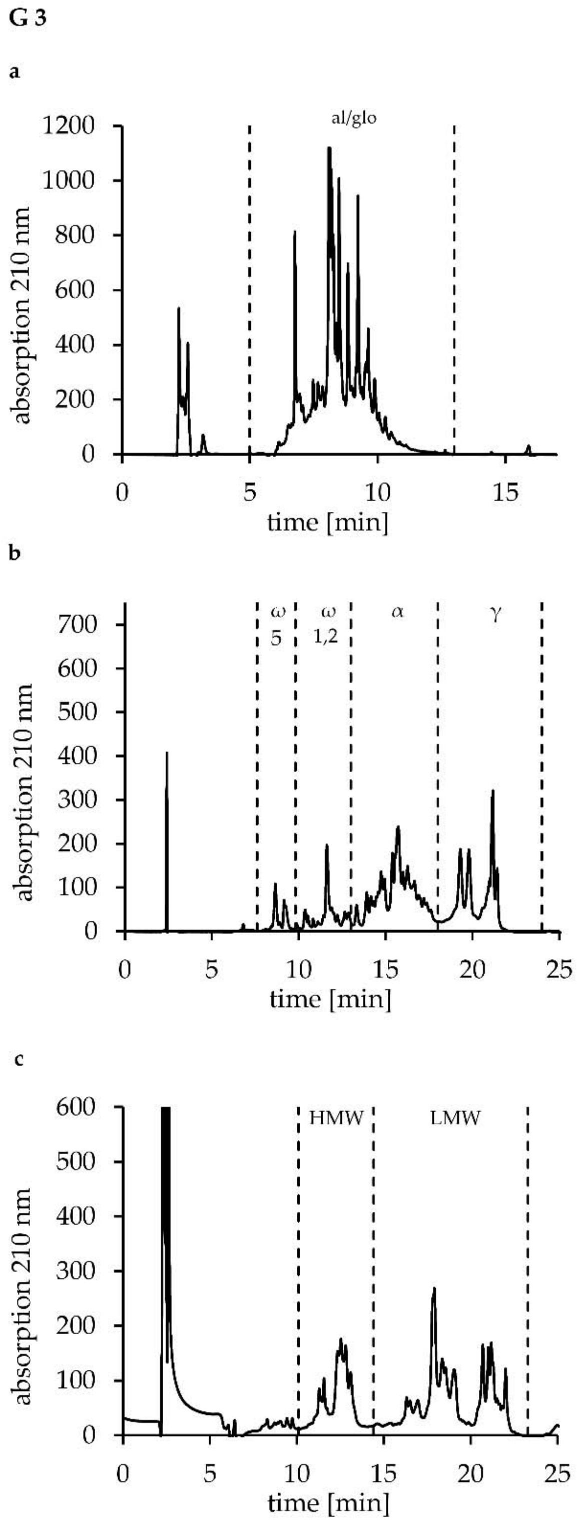

Supplementary Figure S2. Reversed-phase HPLC analysis of G3. Fraction A, albumins and globulins, soluble in aqueous salt solution (**a**), fraction B, gliadins soluble in 60% aqueous ethanol and subdivided into ω5-, ω1,2-, α- and γ-gliadins (**b**) and fraction C, glutenins, soluble in glutenin extraction solution subdivided into high-molecular-weight- (HMW-) and low-molecular-weight- (LMW-) glutenin subunits (**c**).

G 4

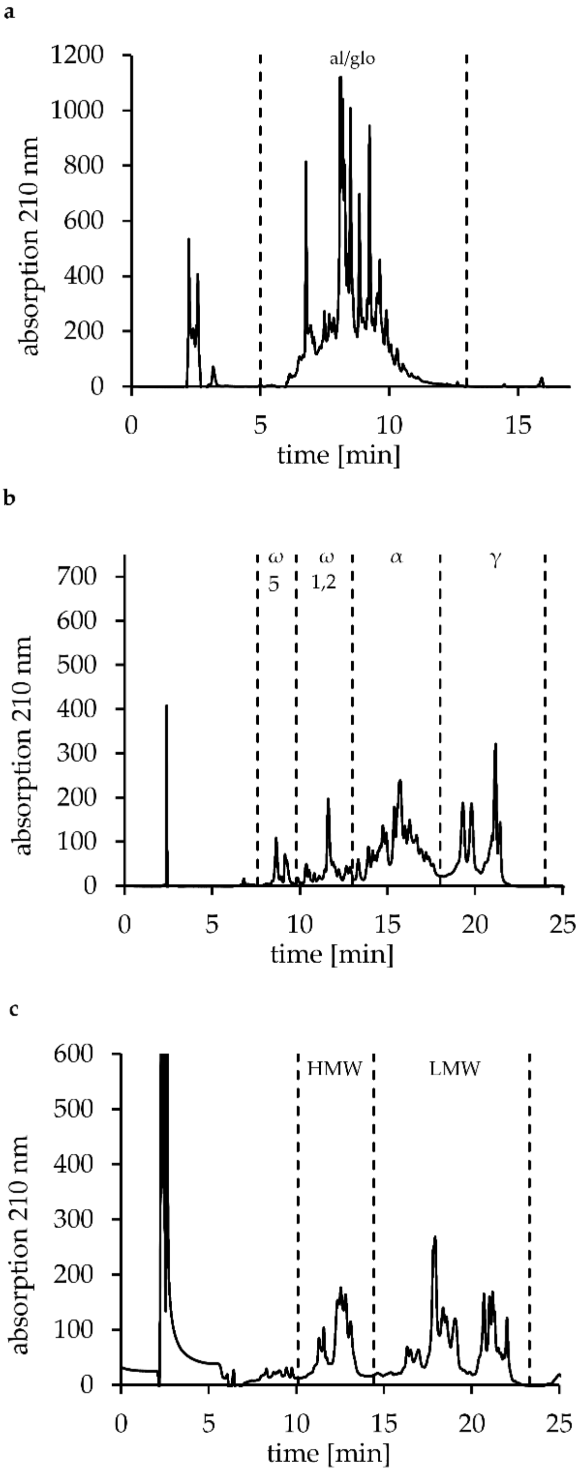

Supplementary Figure S3. Reversed-phase HPLC analysis of G4. Fraction A, albumins and globulins, soluble in aqueous salt solution (**a**), fraction B, gliadins soluble in 60% aqueous ethanol and subdivided into ω5-, ω1,2-, α- and γ-gliadins (**b**) and fraction C, glutenins, soluble in glutenin extraction solution subdivided into high-molecular-weight- (HMW-) and low-molecular-weight- (LMW-) glutenin subunits (**c**).

G 5

a

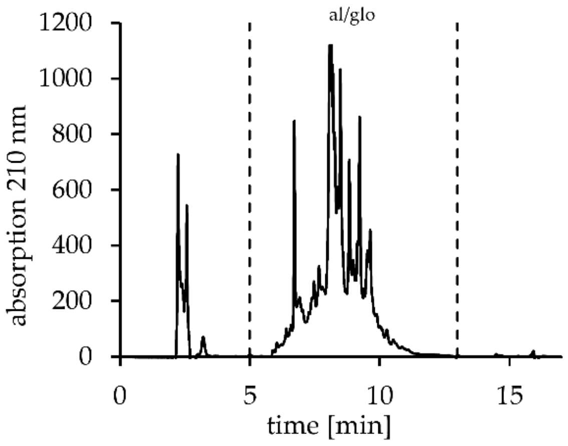

b

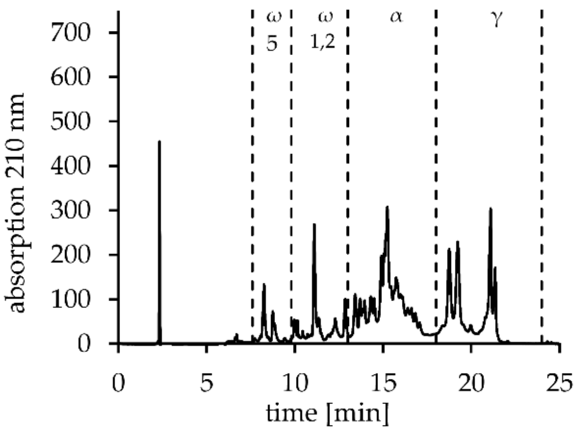

c

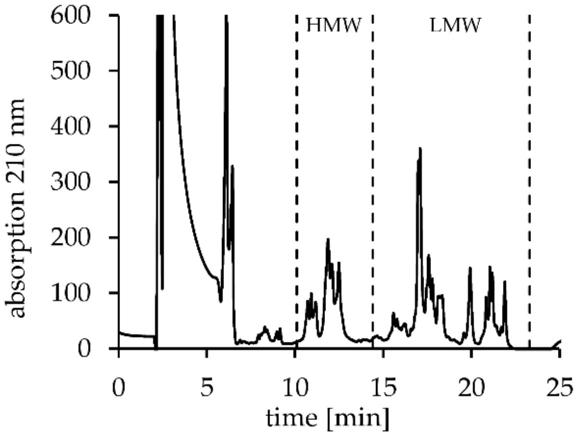

Supplementary Figure S4. Reversed-phase HPLC analysis of G5. Fraction A, albumins and globulins, soluble in aqueous salt solution (**a**), fraction B, gliadins soluble in 60% aqueous ethanol and subdivided into ω5-, ω1,2-, α- and γ-gliadins (**b**) and fraction C, glutenins, soluble in glutenin extraction solution subdivided into high-molecular-weight- (HMW-) and low-molecular-weight- (LMW-) glutenin subunits (**c**).

G 6

a

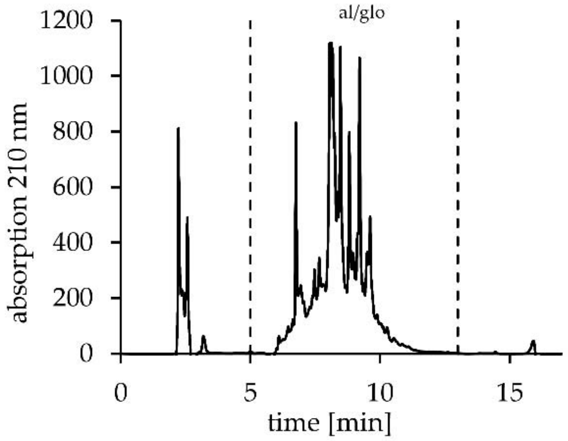

b

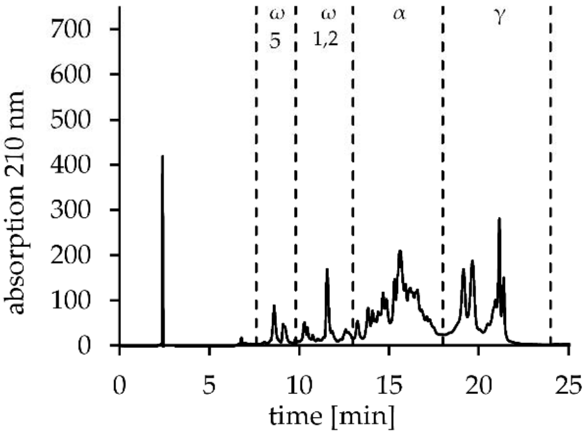

c

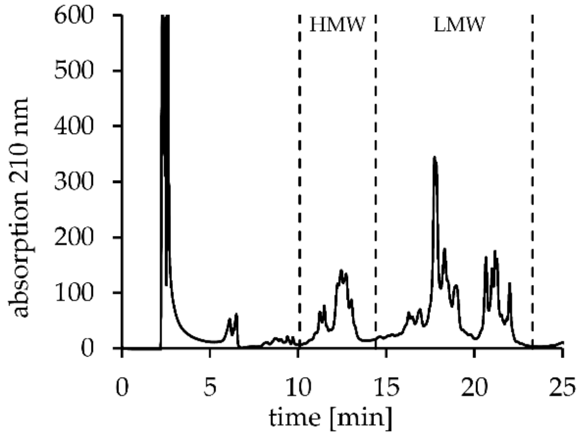

Supplementary Figure S5. Reversed-phase HPLC analysis of G6. Fraction A, albumins and globulins, soluble in aqueous salt solution (**a**), fraction B, gliadins soluble in 60% aqueous ethanol and subdivided into ω5-, ω1,2-, α- and γ-gliadins (**b**) and fraction C, glutenins, soluble in glutenin extraction solution subdivided into high-molecular-weight- (HMW-) and low-molecular-weight- (LMW-) glutenin subunits (**c**).

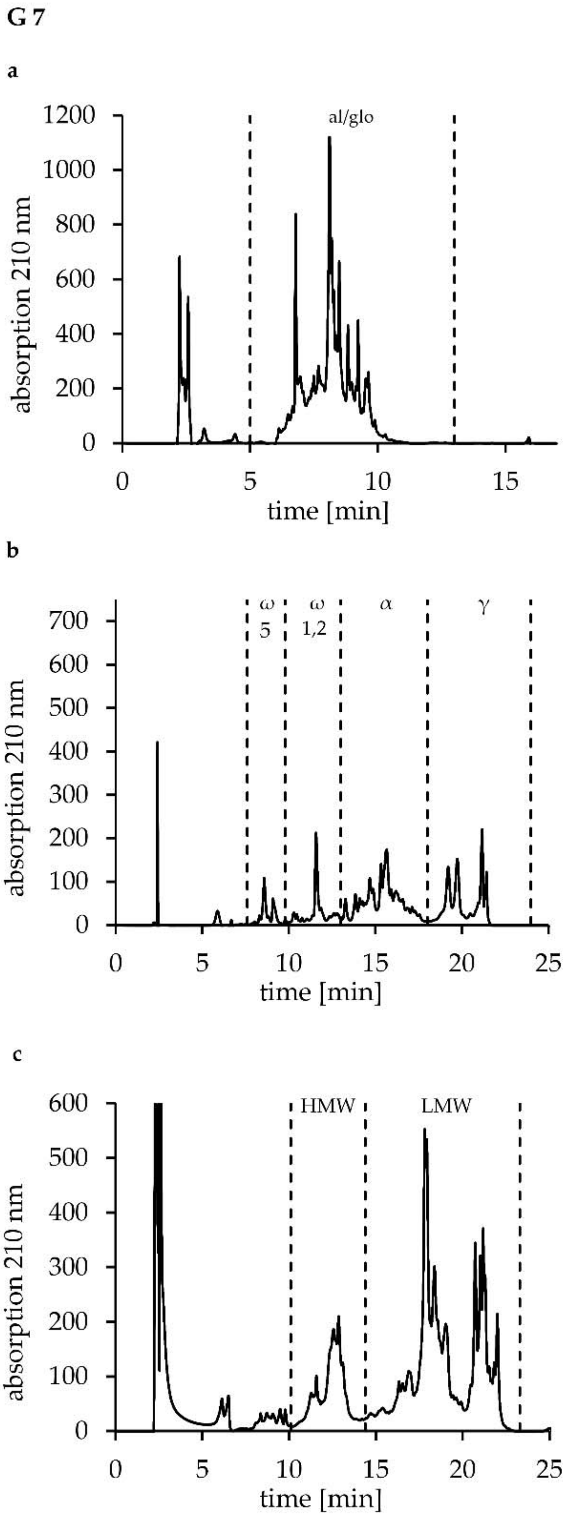

Supplementary Figure S6. Reversed-phase HPLC analysis of G7. Fraction A, albumins and globulins, soluble in aqueous salt solution (**a**), fraction B, gliadins soluble in 60% aqueous ethanol and subdivided into ω5-, ω1,2-, α- and γ-gliadins (**b**) and fraction C, glutenins, soluble in glutenin extraction solution subdivided into high-molecular-weight- (HMW-) and low-molecular-weight- (LMW-) glutenin subunits (**c**).

G 8

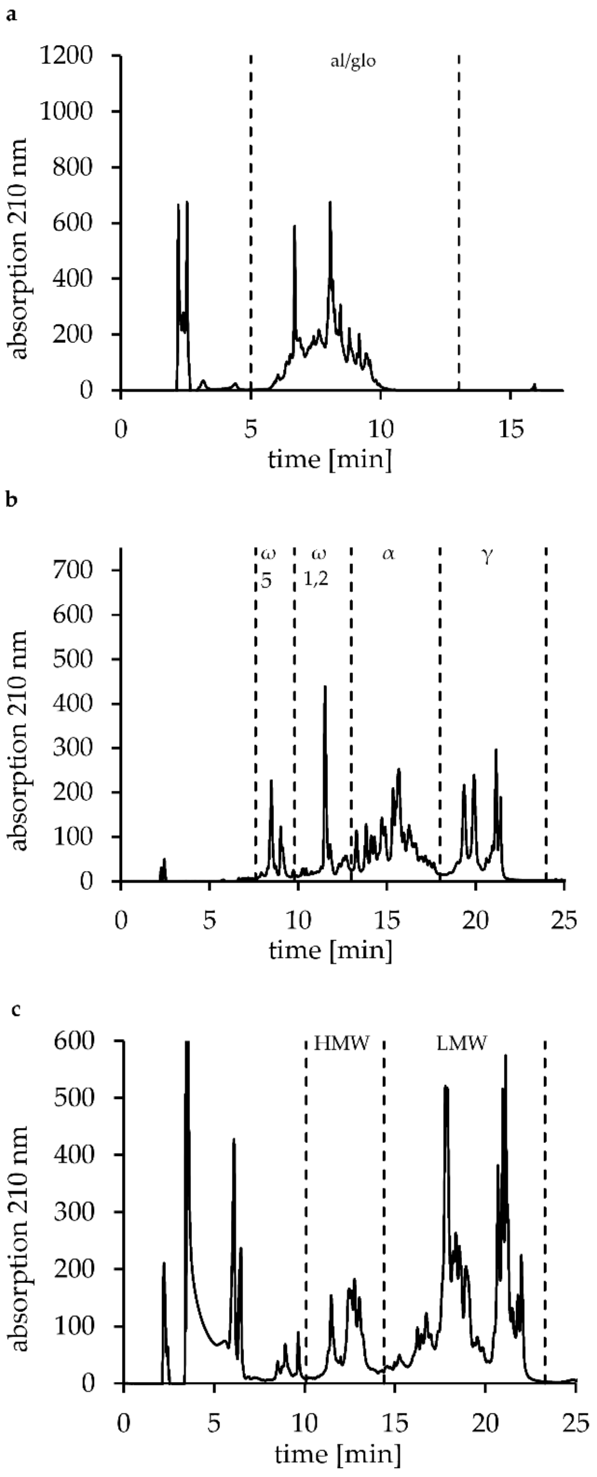

Supplementary Figure S7. Reversed-phase HPLC analysis of G8. Fraction A, albumins and globulins, soluble in aqueous salt solution (**a**), fraction B, gliadins soluble in 60% aqueous ethanol and subdivided into ω5-, ω1,2-, α- and γ-gliadins (**b**) and fraction C, glutenins, soluble in glutenin extraction solution subdivided into high-molecular-weight- (HMW-) and low-molecular-weight- (LMW-) glutenin subunits (**c**).

HWP 1

a

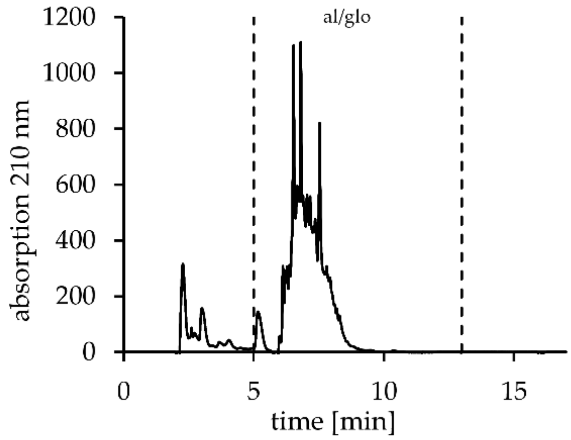

b

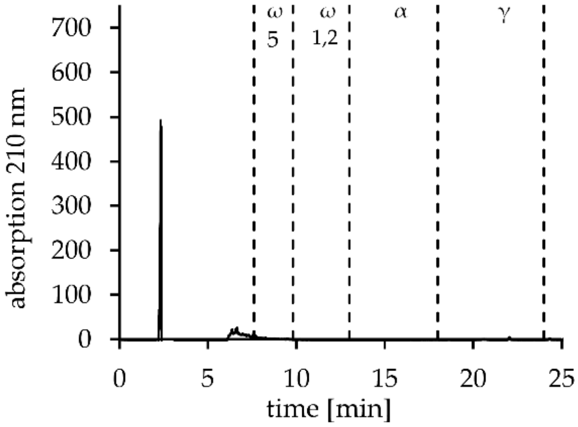

c

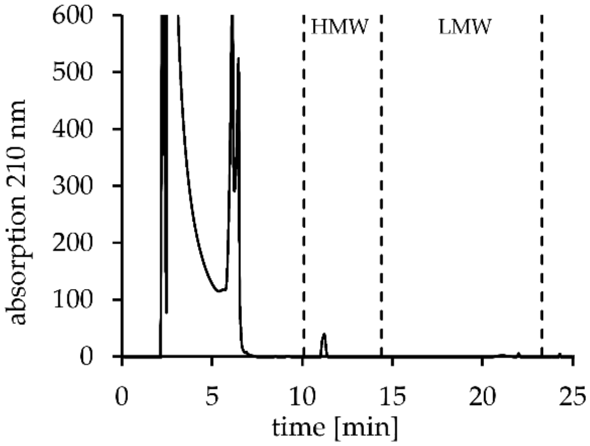

Supplementary Figure S8. Reversed-phase HPLC analysis of HWP1. Fraction A, albumins and globulins, soluble in aqueous salt solution (**a**), fraction B, gliadins soluble in 60% aqueous ethanol and subdivided into ω5-, ω1,2-, α- and γ-gliadins (**b**) and fraction C, glutenins, soluble in glutenin extraction solution subdivided into high-molecular-weight- (HMW-) and low-molecular-weight- (LMW-) glutenin subunits (**c**).

HWP 3

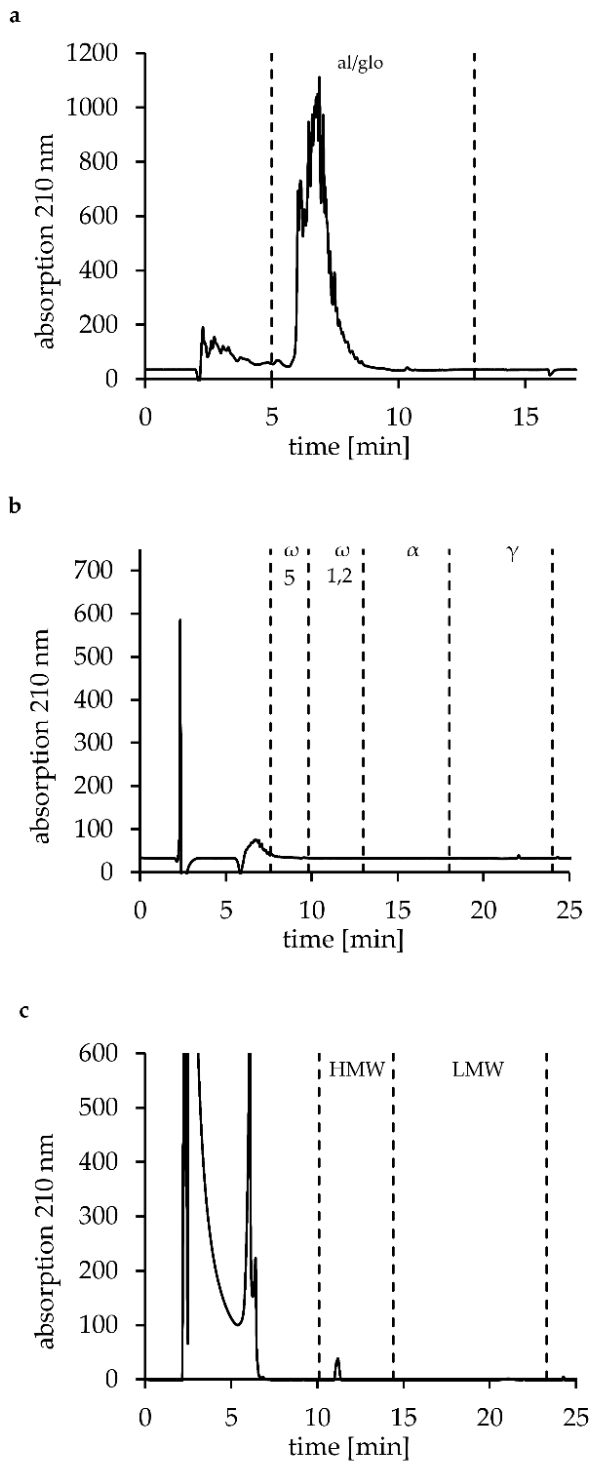

Supplementary Figure S9. Reversed-phase HPLC analysis of HWP3. Fraction A, albumins and globulins, soluble in aqueous salt solution (**a**), fraction B, gliadins soluble in 60% aqueous ethanol and subdivided into ω5-, ω1,2-, α- and γ-gliadins (**b**) and fraction C, glutenins, soluble in glutenin extraction solution subdivided into high-molecular-weight- (HMW-) and low-molecular-weight- (LMW-) glutenin subunits (**c**).

HWP 4

a

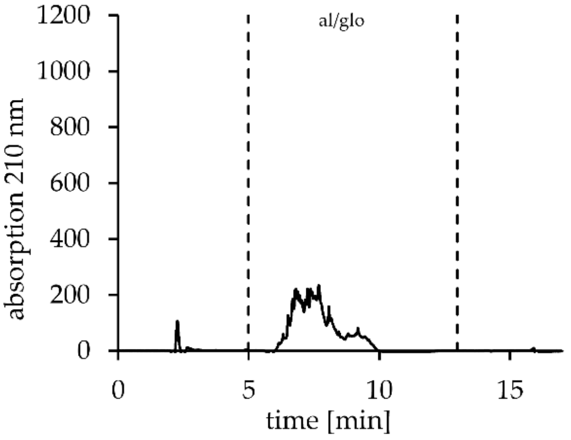

b

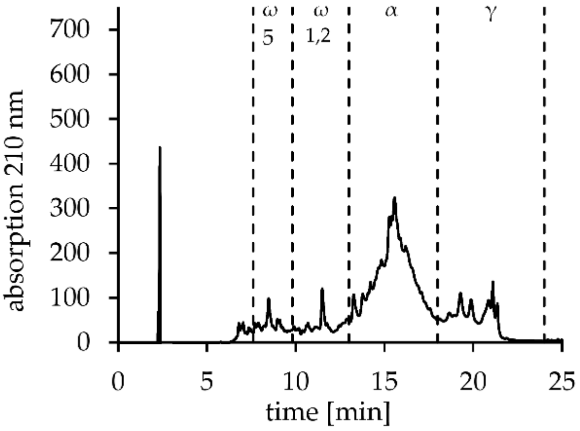

c

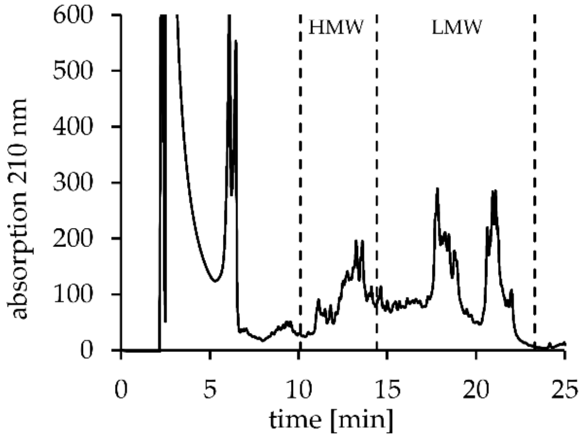

Supplementary Figure S10. Reversed-phase HPLC analysis of HWP4. Fraction A, albumins and globulins, soluble in aqueous salt solution (**a**), fraction B, gliadins soluble in 60% aqueous ethanol and subdivided into ω5-, ω1,2-, α- and γ-gliadins (**b**) and fraction C, glutenins, soluble in glutenin extraction solution subdivided into high-molecular-weight- (HMW-) and low-molecular-weight- (LMW-) glutenin subunits (**c**).

HWP 5

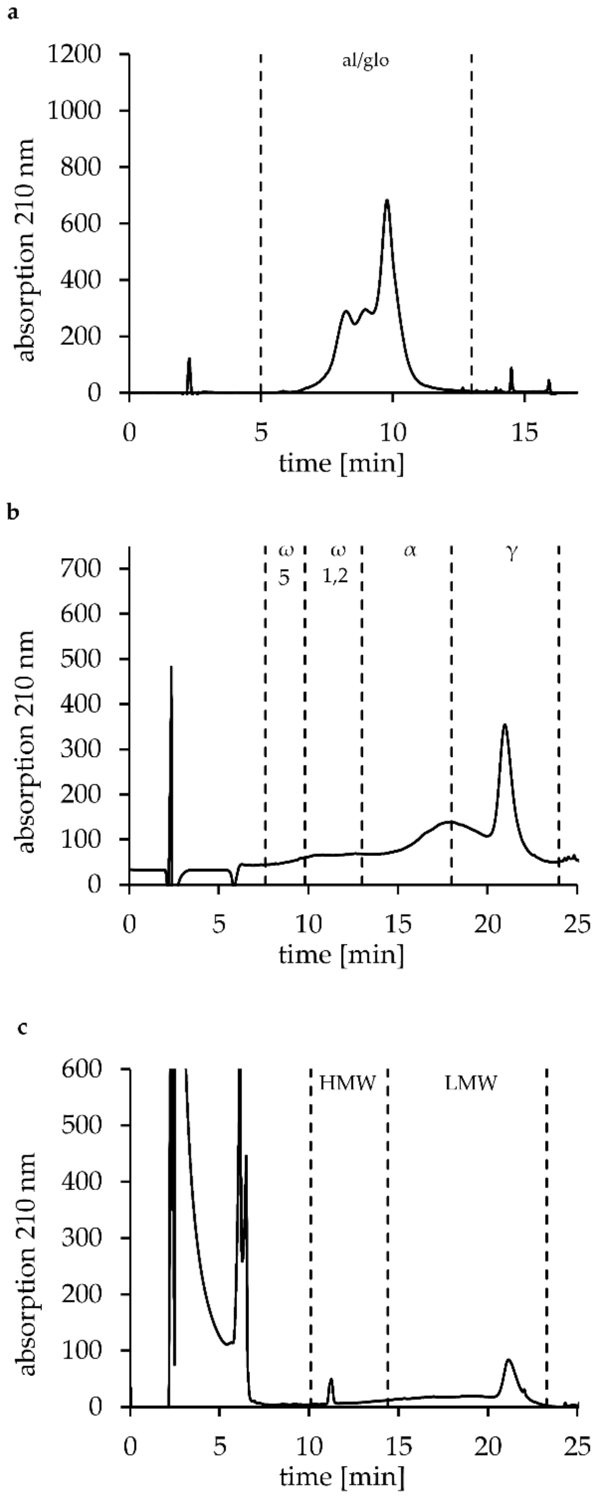

Supplementary Figure S11. Reversed-phase HPLC analysis of HWP5. Fraction A, albumins and globulins, soluble in aqueous salt solution (**a**), fraction B, gliadins soluble in 60% aqueous ethanol and subdivided into ω5-, ω1,2-, α- and γ-gliadins (**b**) and fraction C, glutenins, soluble in glutenin extraction solution subdivided into high-molecular-weight- (HMW-) and low-molecular-weight- (LMW-) glutenin subunits (**c**).

HWP 6

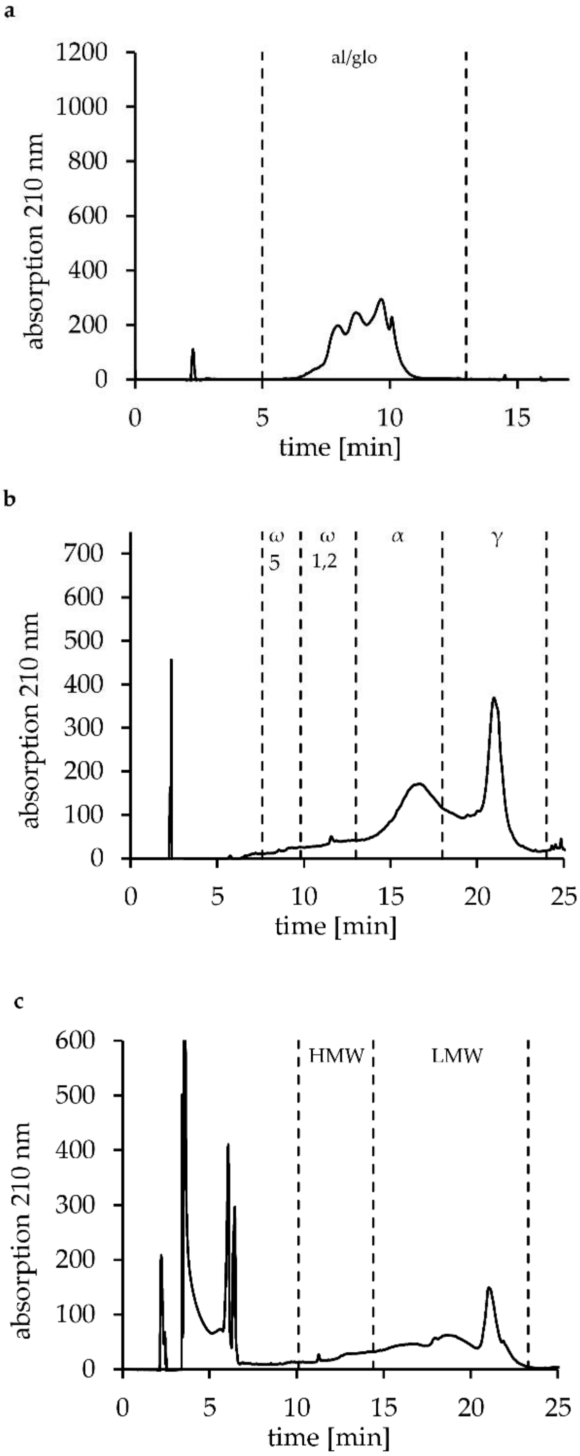

Supplementary Figure S12. Reversed-phase HPLC analysis of HWP6. Fraction A, albumins and globulins, soluble in aqueous salt solution (**a**), fraction B, gliadins soluble in 60% aqueous ethanol and subdivided into ω5-, ω1,2-, α- and γ-gliadins (**b**) and fraction C, glutenins, soluble in glutenin extraction solution subdivided into high-molecular-weight- (HMW-) and low-molecular-weight- (LMW-) glutenin subunits (**c**).

HWP 7

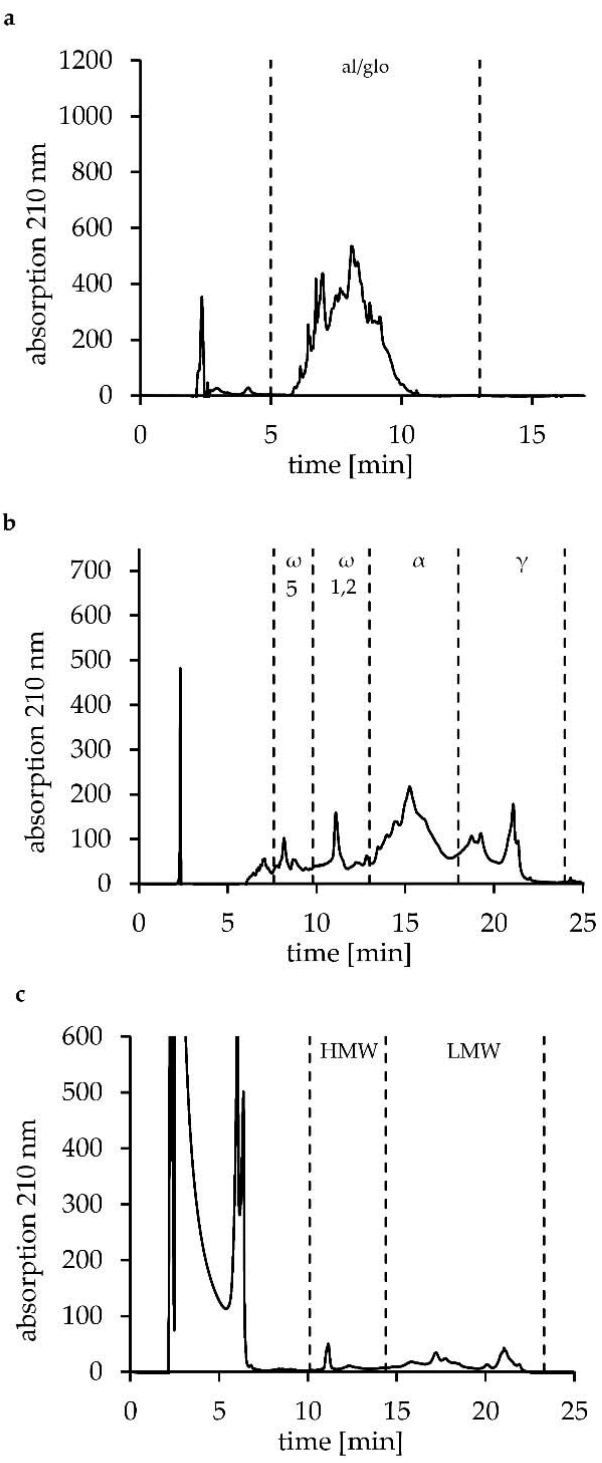

Supplementary Figure S13. Reversed-phase HPLC analysis of HWP7. Fraction A, albumins and globulins, soluble in aqueous salt solution (**a**), fraction B, gliadins soluble in 60% aqueous ethanol and subdivided into ω5-, ω1,2-, α- and γ-gliadins (**b**) and fraction C, glutenins, soluble in glutenin extraction solution subdivided into high-molecular-weight- (HMW-) and low-molecular-weight- (LMW-) glutenin subunits (**c**).

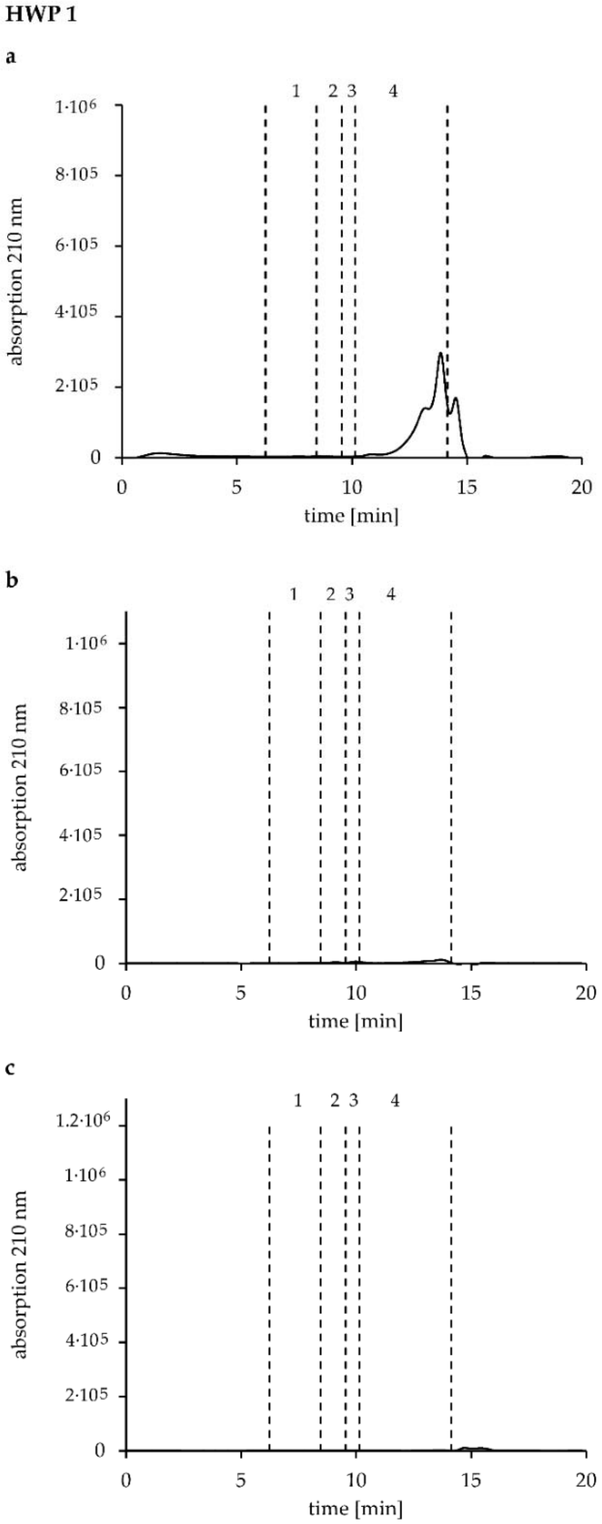

Supplementary Figure S14. Gel-permeation HPLC analysis of HWP1. Fraction I, soluble in aqueous salt solution (**a**), fraction II, soluble in 60% aqueous ethanol (**b**) and fraction III, soluble in acetonitrile/water (50/50, v/v) (**c**) analyzed using system I with the following ranges of relative molecular masses: M_r 200-66 kDa (1), M_r 66-29 kDa (2), 29-14 kDa (3), <14 kDa (4).

HWP 3

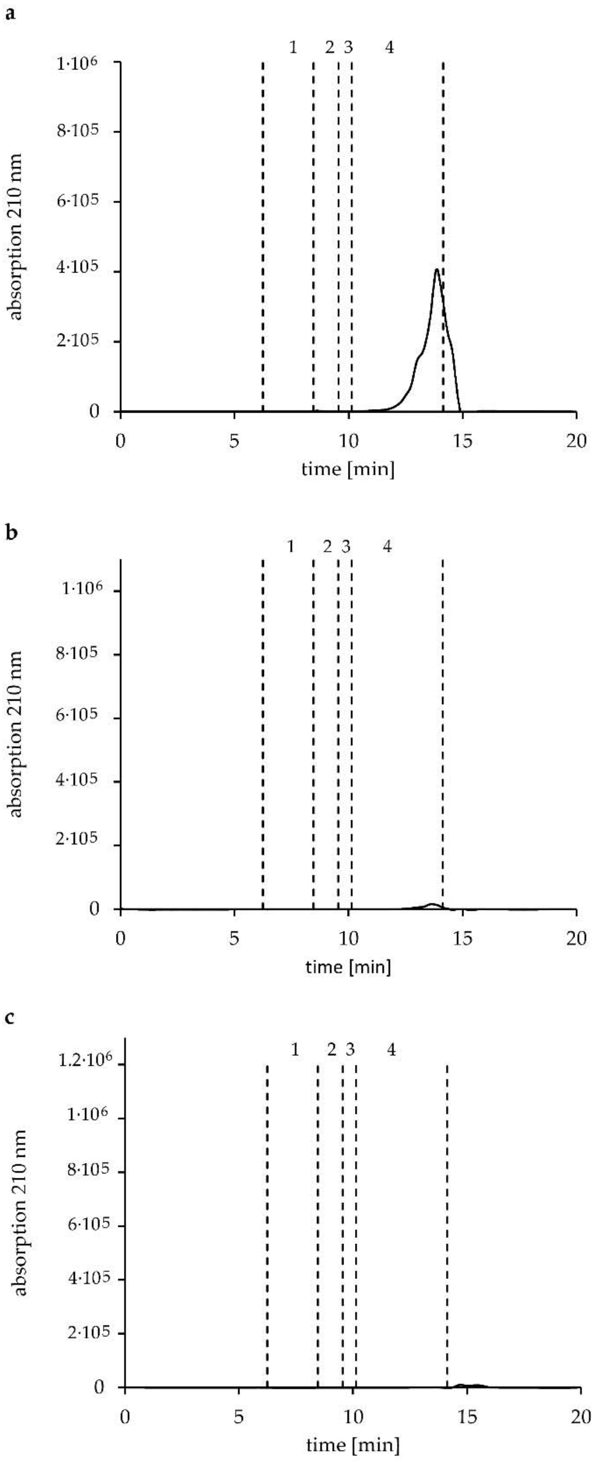

Supplementary Figure S15. Gel-permeation HPLC analysis of HWP3. Fraction I, soluble in aqueous salt solution (a), fraction II, soluble in 60% aqueous ethanol (b) and fraction III, soluble in acetonitrile/water (50/50, v/v) (c) analyzed using system I with the following ranges of relative molecular masses: M_r 200-66 kDa (1), M_r 66-29 kDa (2), 29-14 kDa (3), <14 kDa (4).

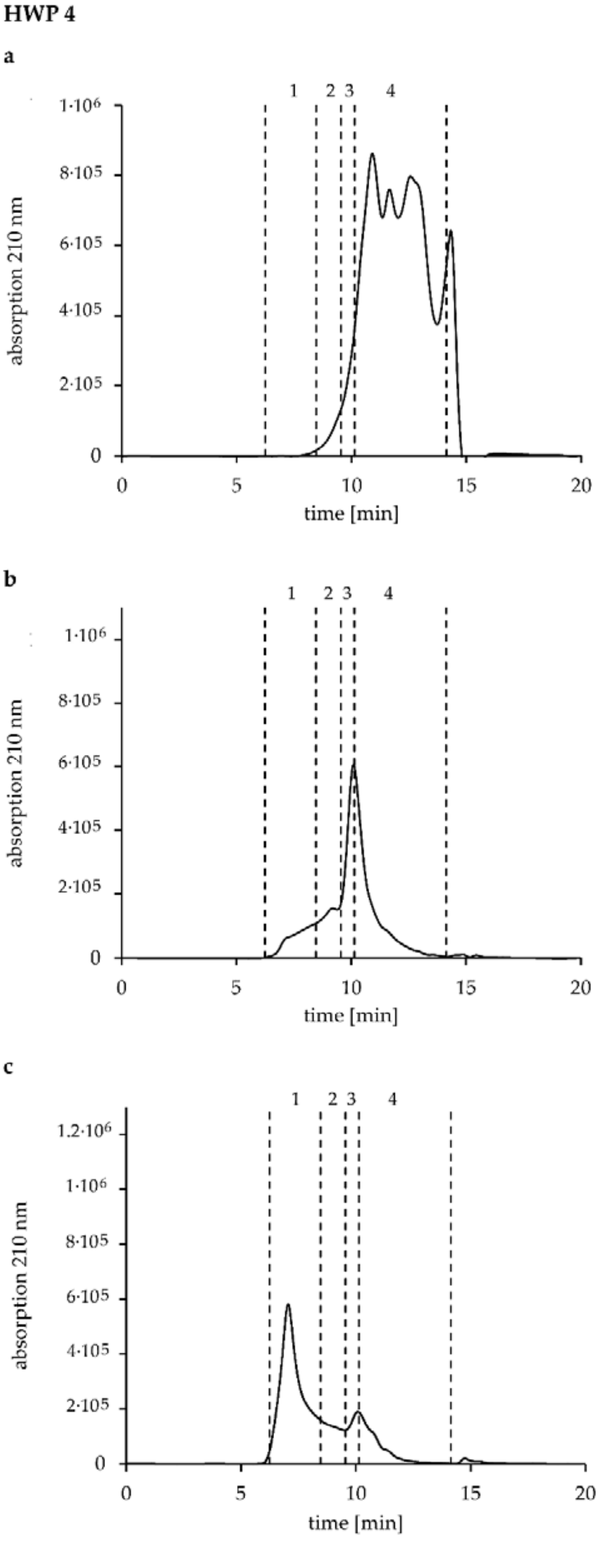

Supplementary Figure S16. Gel-permeation HPLC analysis of HWP4. Fraction I, soluble in aqueous salt solution (a), fraction II, soluble in 60% aqueous ethanol (b) and fraction III, soluble in acetonitrile/water (50/50, v/v) (c) analyzed using system I with the following ranges of relative molecular masses: M_r 200-66 kDa (1), M_r 66-29 kDa (2), 29-14 kDa (3), <14 kDa (4).

HWP 5

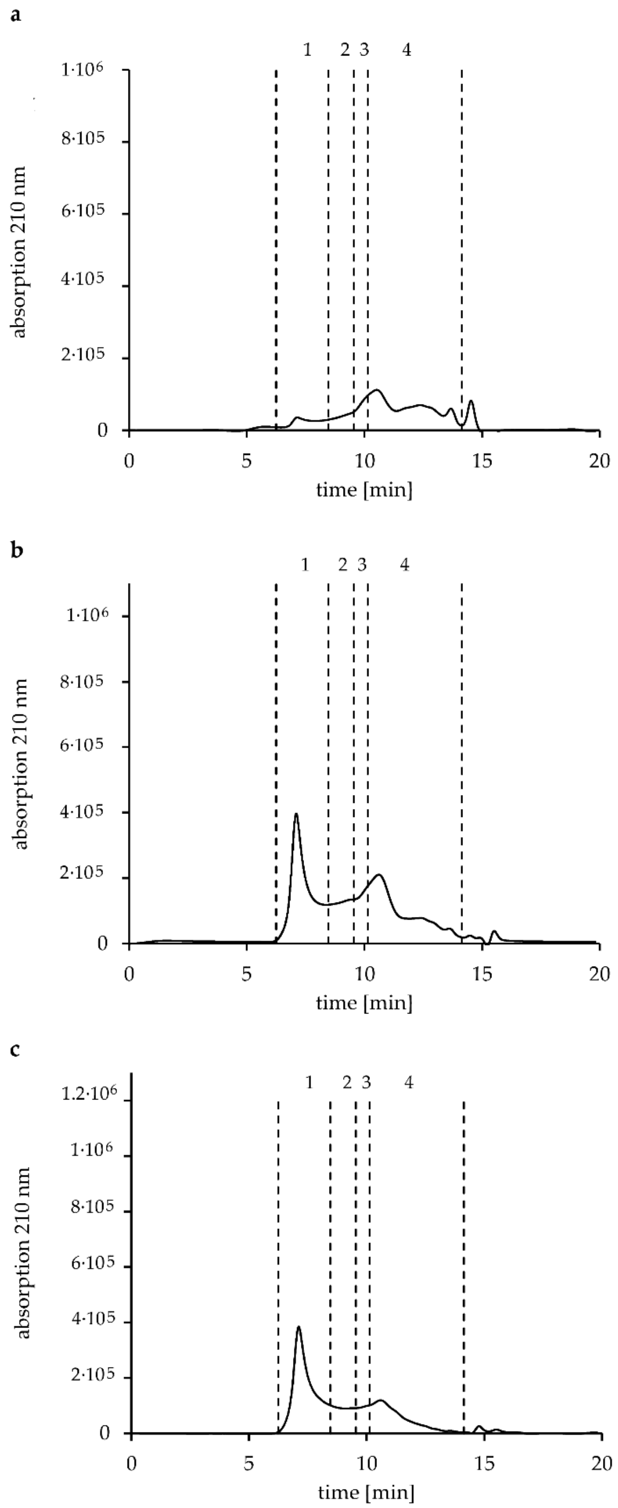

Supplementary Figure S17. Gel-permeation HPLC analysis of HWP5. Fraction I, soluble in aqueous salt solution (a), fraction II, soluble in 60% aqueous ethanol (b) and fraction III, soluble in acetonitrile/water (50/50, v/v) (c) analyzed using system I with the following ranges of relative molecular masses: M_r 200-66 kDa (1), M_r 66-29 kDa (2), 29-14 kDa (3), <14 kDa (4).

HWP 6

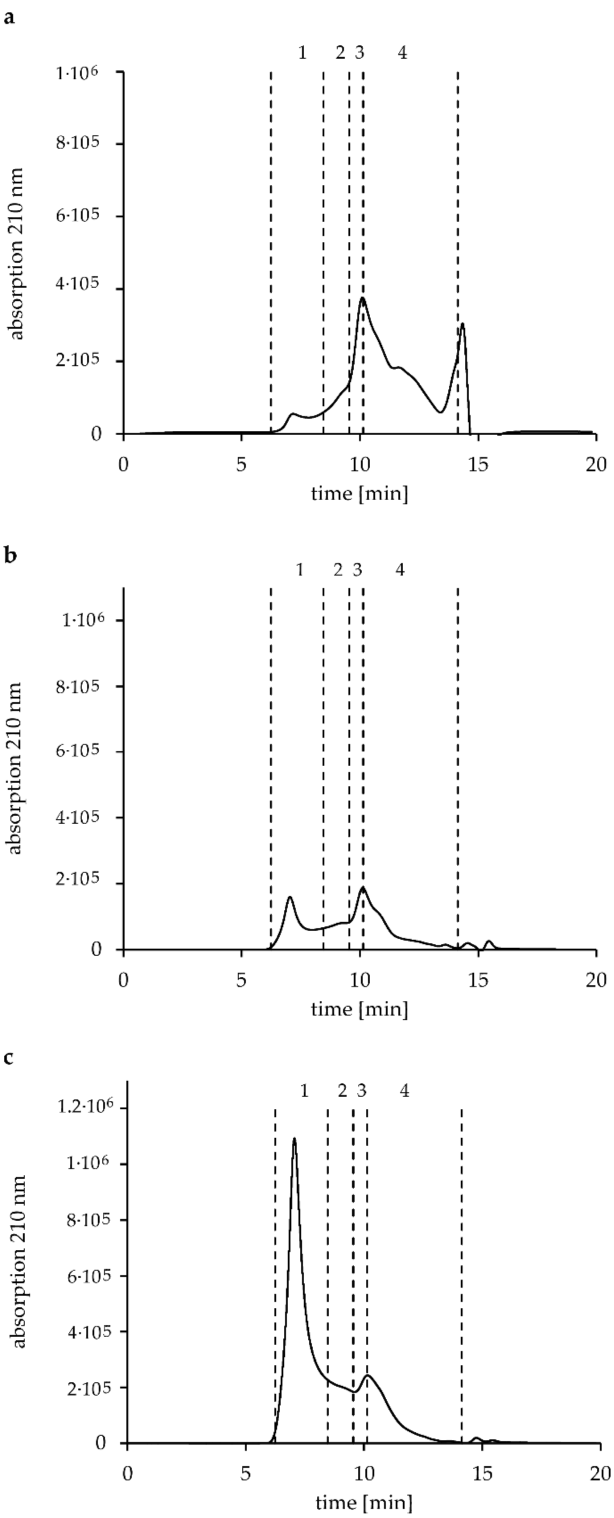

Supplementary Figure S18. Gel-permeation HPLC analysis of HWP6. Fraction I, soluble in aqueous salt solution (a), fraction II, soluble in 60% aqueous ethanol (b) and fraction III, soluble in acetonitrile/water (50/50, v/v) (c) analyzed using system I with the following ranges of relative molecular masses: M_r 200-66 kDa (1), M_r 66-29 kDa (2), 29-14 kDa (3), <14 kDa (4).

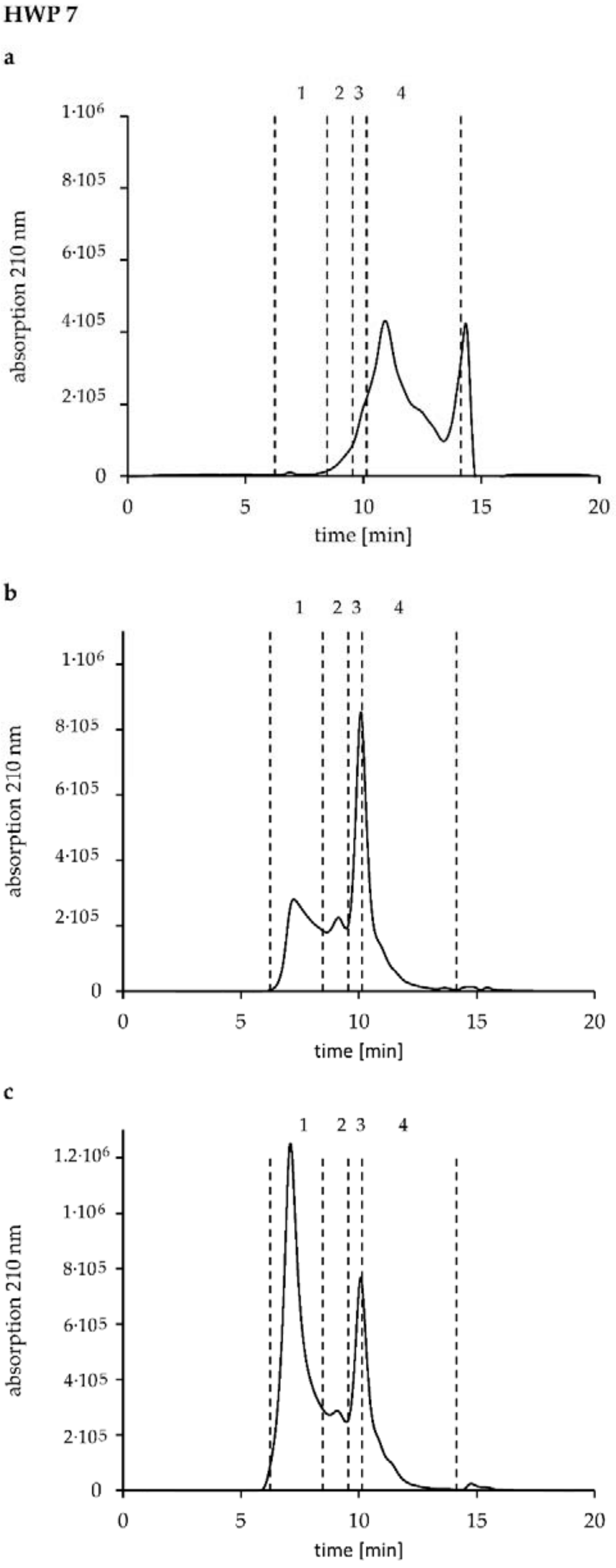

Supplementary Figure S19. Gel-permeation HPLC analysis of HWP7. Fraction I, soluble in aqueous salt solution (a), fraction II, soluble in 60% aqueous ethanol (b) and fraction III, soluble in acetonitrile/water (50/50, v/v) (c) analyzed using system I with the following ranges of relative molecular masses: M_r 200-66 kDa (1), M_r 66-29 kDa (2), 29-14 kDa (3), <14 kDa (4).

G 7

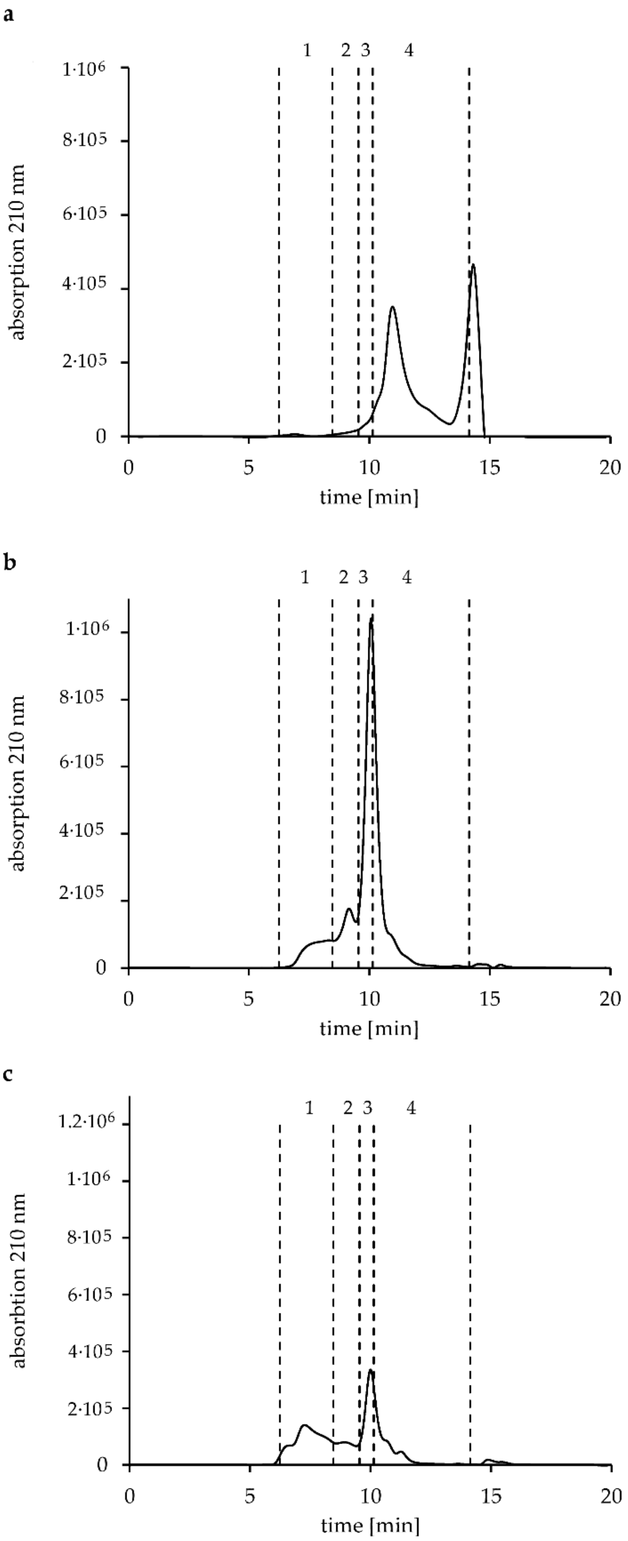

Supplementary Figure S20. Gel-permeation HPLC analysis of G7. Fraction I, soluble in aqueous salt solution (a), fraction II, soluble in 60% aqueous ethanol (b) and fraction III, soluble in acetonitrile/water (50/50, v/v) (c) analyzed using system I with the following ranges of relative molecular masses: M_r 200-66 kDa (1), M_r 66-29 kDa (2), 29-14 kDa (3), <14 kDa (4).

G 8

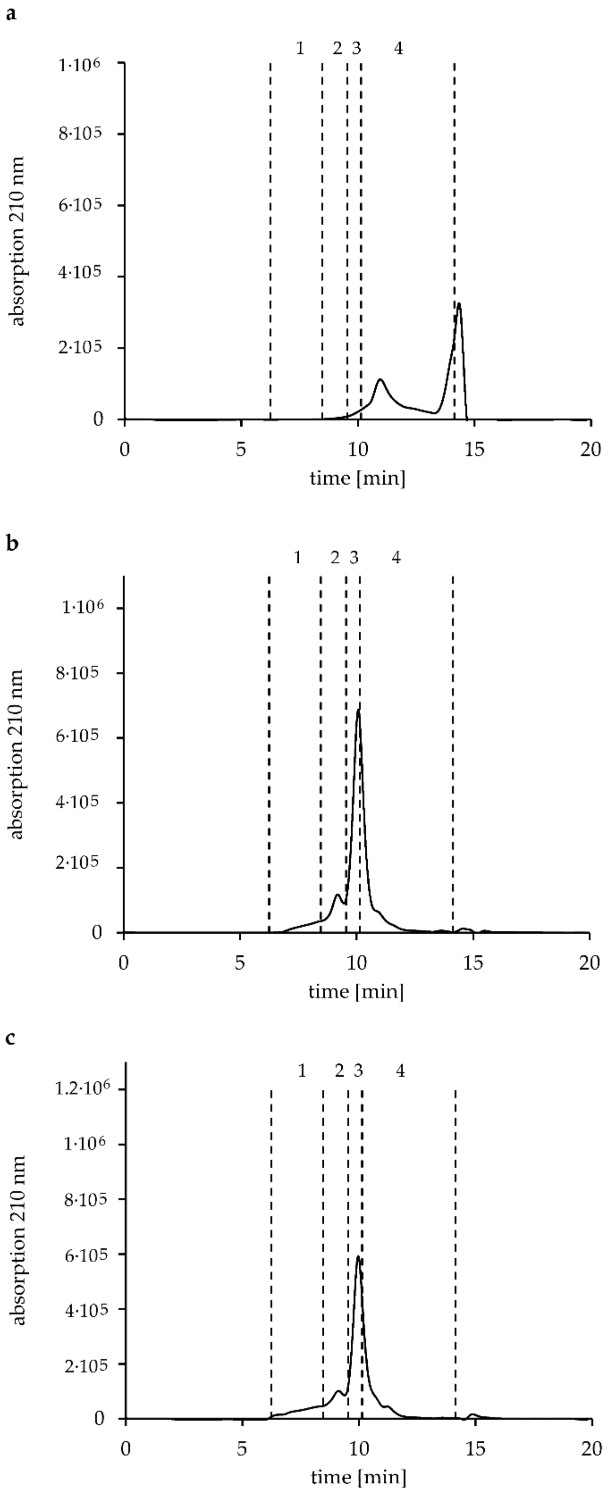

Supplementary Figure S21. Gel-permeation HPLC analysis of G8. Fraction I, soluble in aqueous salt solution (a), fraction II, soluble in 60% aqueous ethanol (b) and fraction III, soluble in acetonitrile/water (50/50, v/v) (c) analyzed using system I with the following ranges of relative molecular masses: M_r 200-66 kDa (1), M_r 66-29 kDa (2), 29-14 kDa (3), <14 kDa (4).

HWP 1

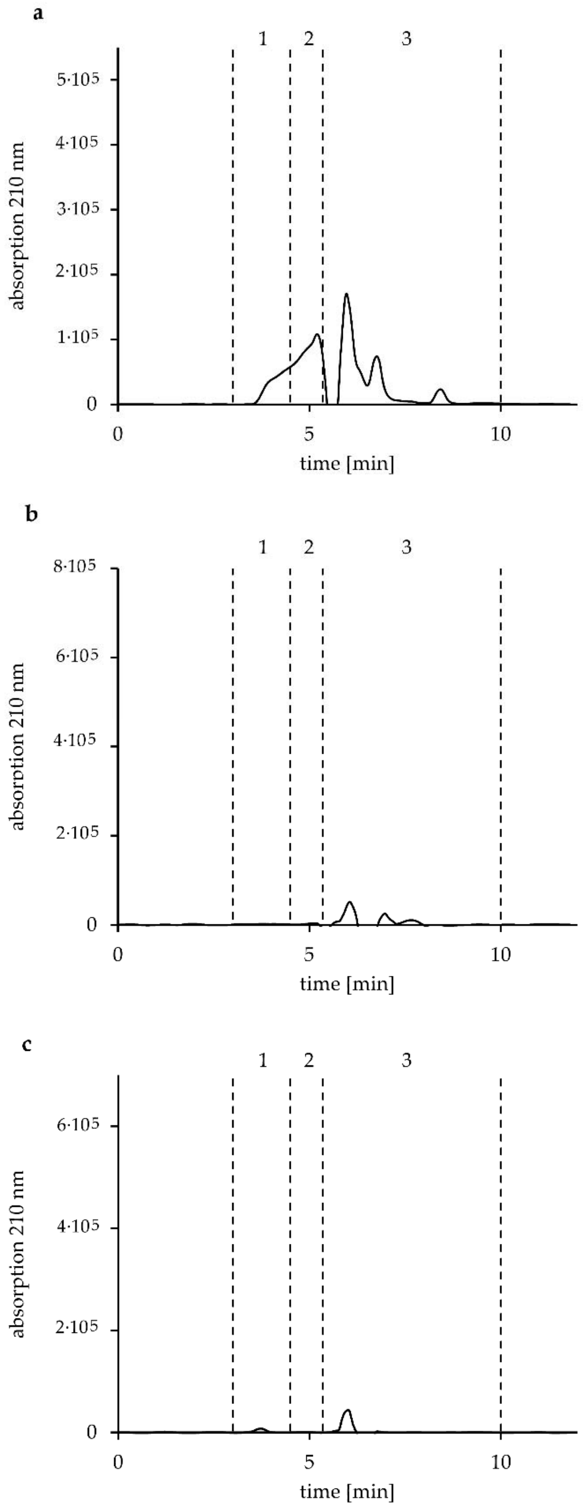

Supplementary Figure S22. Gel-permeation HPLC analysis of HWP1. Fraction II, soluble in aqueous salt solution (a), fraction II, soluble in 60% aqueous ethanol (b) and fraction III, soluble in acetonitrile/water (50/50, v/v) (c) analyzed using system II with the following ranges of relative molecular masses: $M_r \geq 14$ kDa (1), M_r 14-2 kDa (2), $M_r < 2$ kDa (3).

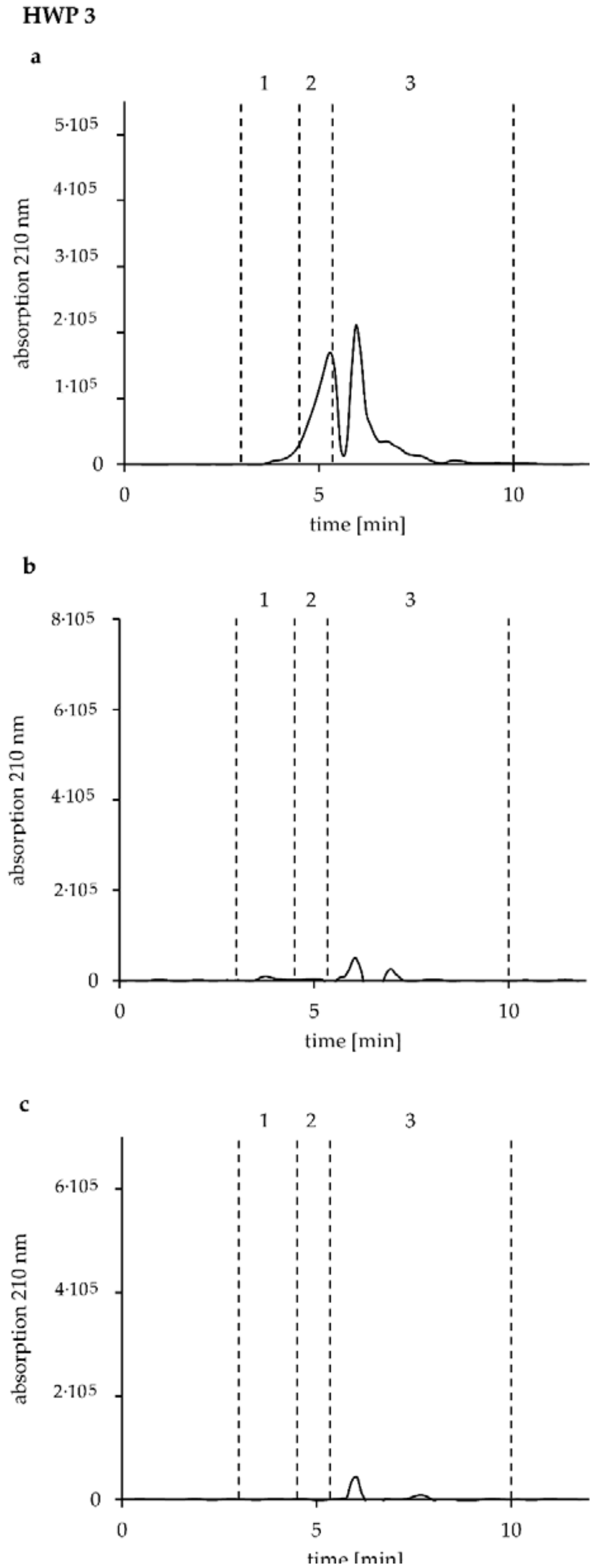

Supplementary Figure S23. Gel-permeation HPLC analysis of HWP3. Fraction II, soluble in aqueous salt solution (a), fraction II, soluble in 60% aqueous ethanol (b) and fraction III, soluble in acetonitrile/water (50/50, v/v) (c) analyzed using system II with the following ranges of relative molecular masses: $M_r \geq 14$ kDa (1), M_r 14-2 kDa (2), $M_r < 2$ kDa (3).

HWP 4

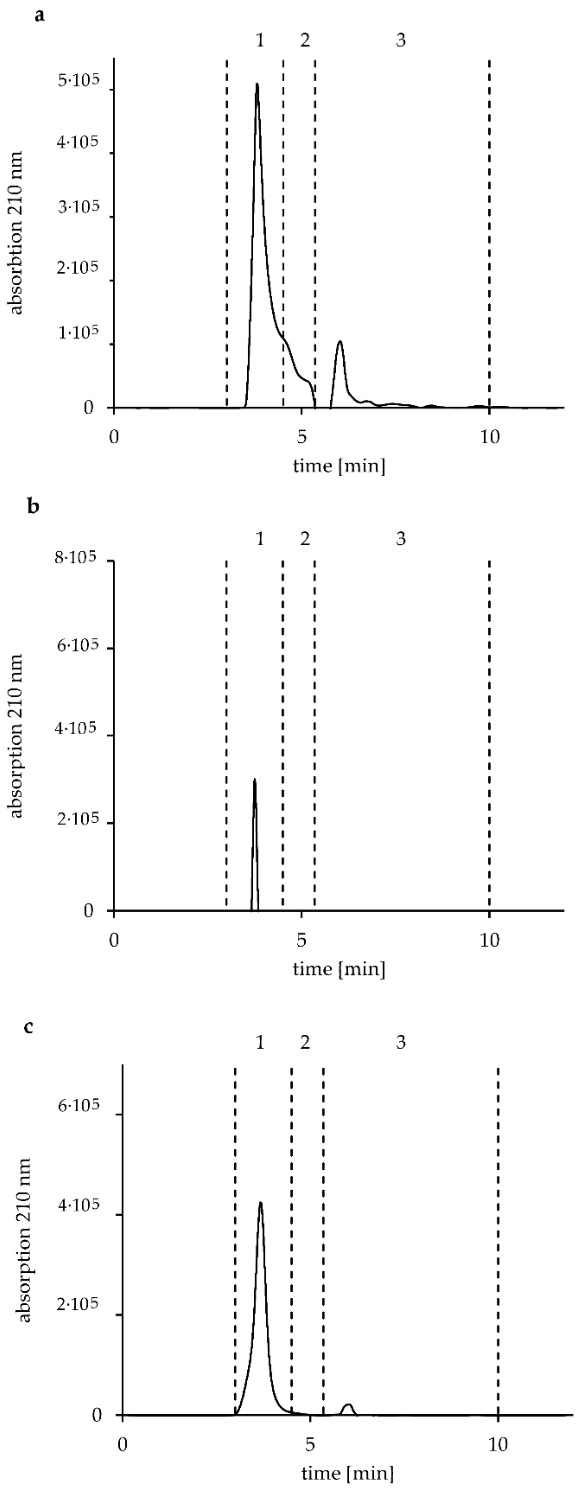

Supplementary Figure S24. Gel-permeation HPLC analysis of HWP4. Fraction II, soluble in aqueous salt solution (a), fraction II, soluble in 60% aqueous ethanol (b) and fraction III, soluble in acetonitrile/water (50/50, v/v) (c) analyzed using system II with the following ranges of relative molecular masses: $M_r \geq 14$ kDa (1), M_r 14-2 kDa (2), $M_r < 2$ kDa (3).

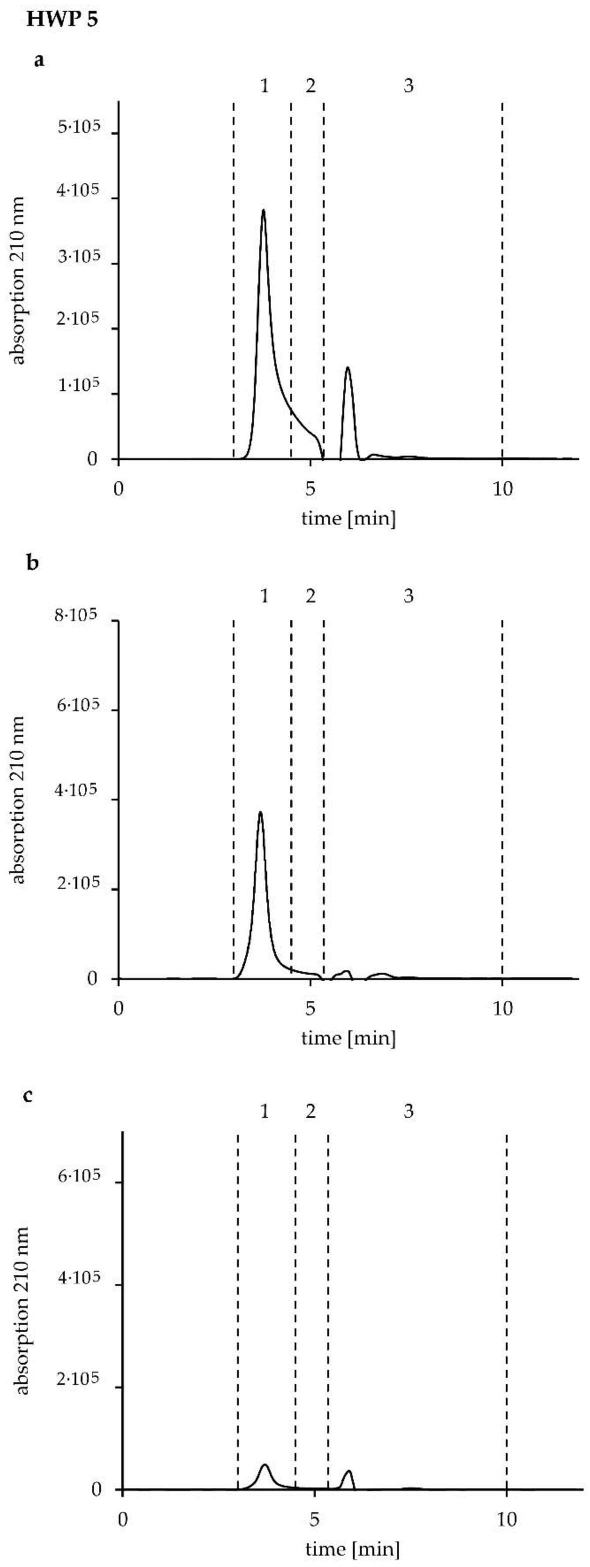

Supplementary Figure S25. Gel-permeation HPLC analysis of HWP5. Fraction II, soluble in aqueous salt solution (a), fraction II, soluble in 60% aqueous ethanol (b) and fraction III, soluble in acetonitrile/water (50/50, v/v) (c) analyzed using system II with the following ranges of relative molecular masses: $M_r \geq 14$ kDa (1), M_r 14-2 kDa (2), $M_r < 2$ kDa (3).

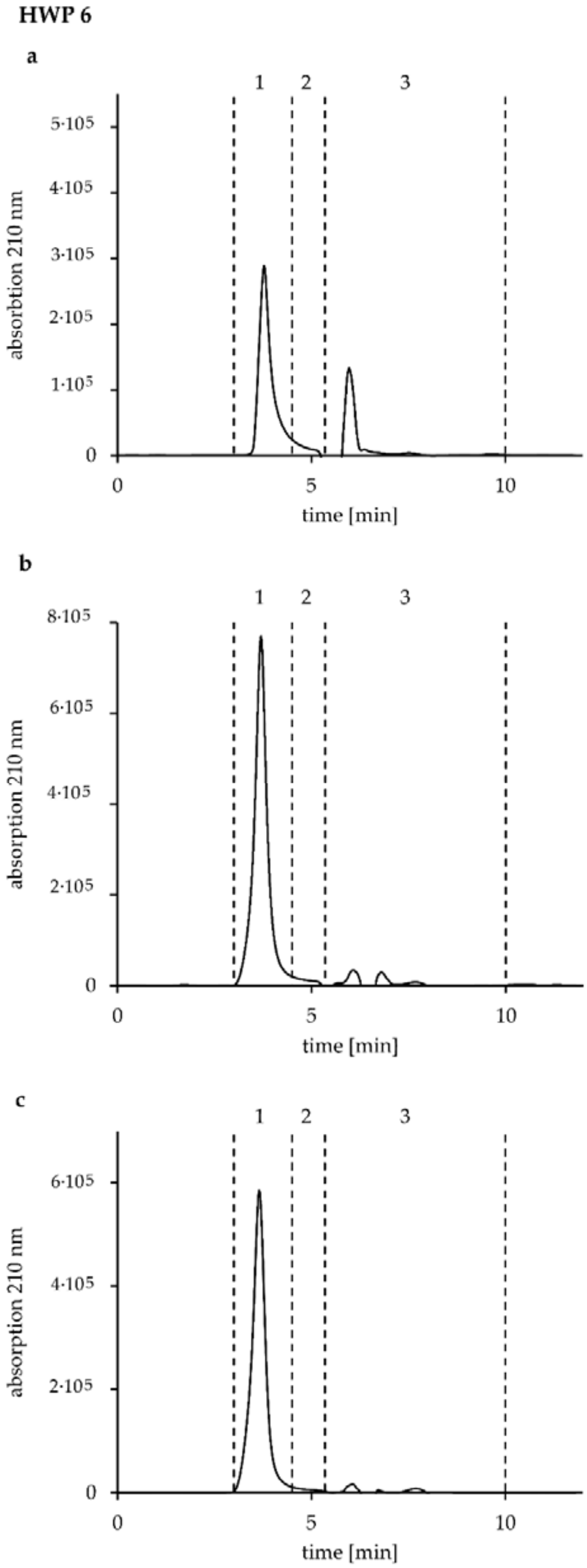

Supplementary Figure S26. Gel-permeation HPLC analysis of HWP6. Fraction II, soluble in aqueous salt solution (a), fraction II, soluble in 60% aqueous ethanol (b) and fraction III, soluble in acetonitrile/water (50/50, v/v) (c) analyzed using system II with the following ranges of relative molecular masses: $M_r \geq 14$ kDa (1), M_r 14-2 kDa (2), $M_r < 2$ kDa (3).

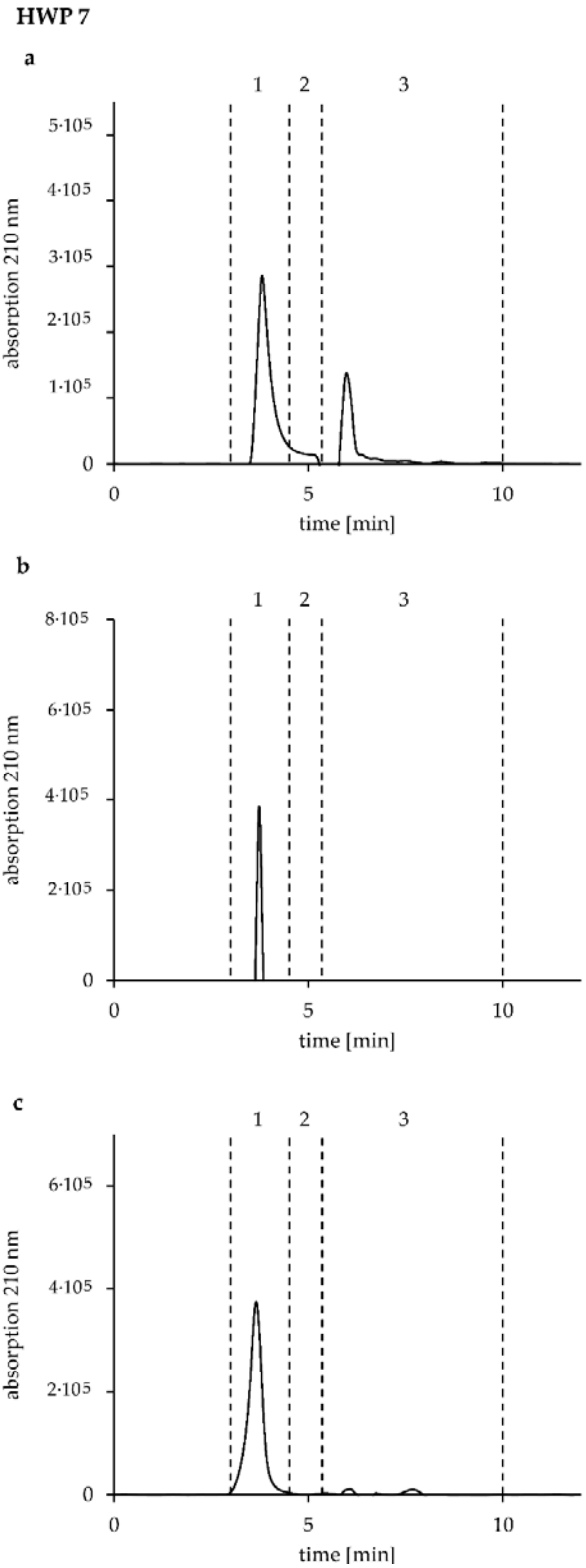

Supplementary Figure S27. Gel-permeation HPLC analysis of HWP7. Fraction II, soluble in aqueous salt solution (a), fraction II, soluble in 60% aqueous ethanol (b) and fraction III, soluble in acetonitrile/water (50/50, v/v) (c) analyzed using system II with the following ranges of relative molecular masses: $M_r \geq 14$ kDa (1), M_r 14-2 kDa (2), $M_r < 2$ kDa (3).

G 7

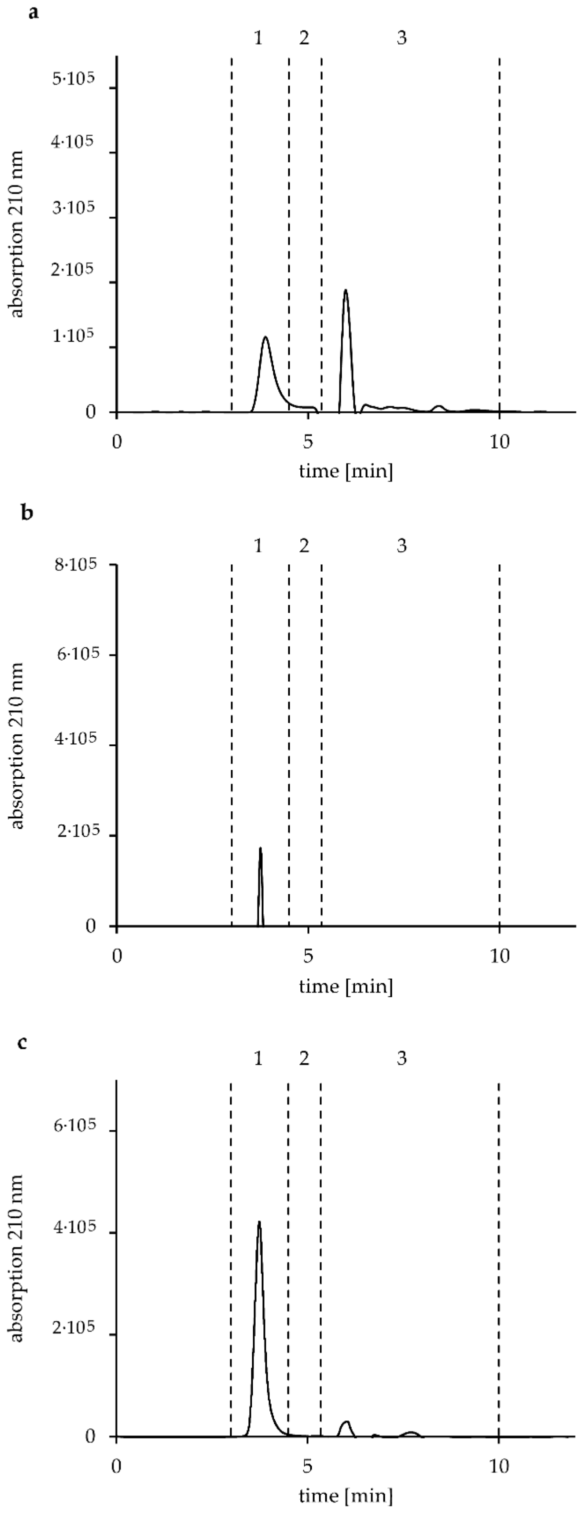

Supplementary Figure S28. Gel-permeation HPLC analysis of G7. Fraction II, soluble in aqueous salt solution (a), fraction II, soluble in 60% aqueous ethanol (b) and fraction III, soluble in acetonitrile/water (50/50, v/v) (c) analyzed using system II with the following ranges of relative molecular masses: $M_r \geq 14$ kDa (1), M_r 14-2 kDa (2), $M_r < 2$ kDa (3).

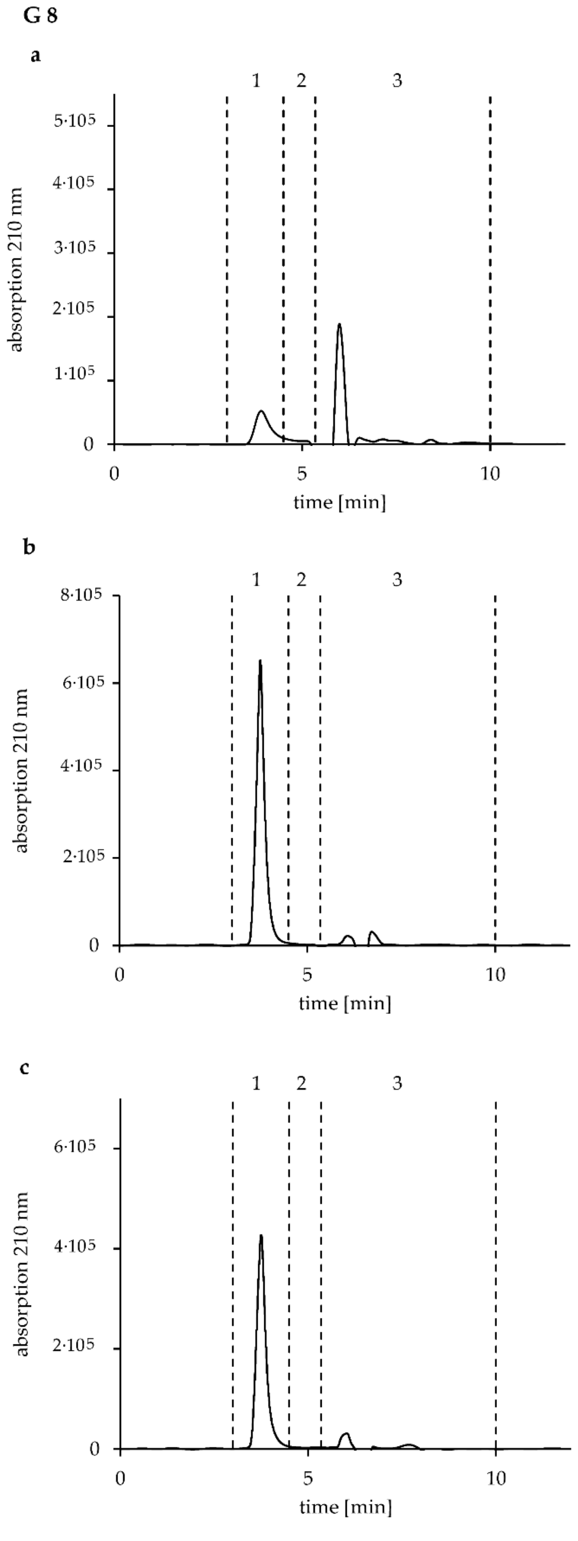

Supplementary Figure S29. Gel-permeation HPLC analysis of G8. Fraction II, soluble in aqueous salt solution (a), fraction II, soluble in 60% aqueous ethanol (b) and fraction III, soluble in acetonitrile/water (50/50, v/v) (c) analyzed using system II with the following ranges of relative molecular masses: $M_r \geq 14$ kDa (1), M_r 14-2 kDa (2), $M_r < 2$ kDa (3).
